# Cardio-Protective Properties and Health Benefits of Fish Lipid Bioactives; The Effects of Thermal Processing

**DOI:** 10.3390/md20030187

**Published:** 2022-03-02

**Authors:** Alexandros Tsoupras, Chloe Brummell, Ciara Kealy, Karolis Vitkaitis, Shane Redfern, Ioannis Zabetakis

**Affiliations:** 1 Department of Biological Sciences, University of Limerick, V94 T9PX Limerick, Ireland; chloebrummell8@gmail.com (C.B.); ciarakealy@icloud.com (C.K.); karolisvitkaitis@yahoo.com (K.V.); ioannis.zabetakis@ul.ie (I.Z.); 2 Health Research Institute, University of Limerick, V94 T9PX Limerick, Ireland; 3 Bernal Institute, University of Limerick, V94 T9PX Limerick, Ireland; 4 Department of Sport, Leisure & Tourism, Limerick Institute of Technology, Moylish Park, V94 E8YF Limerick, Ireland; shane.redfern@lit.ie

**Keywords:** fish, fish oil, lipid bioactives, lipid vitamins, polar lipids, PUFA, carotenoids, antithrombotic, anti-inflammatory, cardio-protective, PAF, thrombin, thermal processing

## Abstract

The beneficial effects of fish-derived lipid bioactives have come to prominence over the last few decades, especially for their utilization in fish oils, supplements, and nutraceuticals. Omega-3 (n-3) polyunsaturated fatty acids (PUFA), lipid vitamins, carotenoids, and polar lipid bioactives from fish have shown to possess a vast range of beneficial effects against a multitude of chronic disorders and especially against inflammation-and cardiovascular disorders (CVD). The observed cardio-protective effects and health benefits are believed to be attributed to the synergy of these fish-derived lipid bioactives. Within the present article the recent findings in the literature on the lipid content of the mainly consumed fish species, their bio-functionality, and cardio-protective benefits is thoroughly reviewed. Moreover, the recovery and valorization of such lipid bioactives from fish by-products and fishing by-catch, in order to reduce waste, while developing useful products containing cardio-protective lipids from the leftover materials of fisheries and aquaculture industries, are also of industrial and environmental interest. Emphasis is also given to the effects of heat treatments during fish processing on the structures and bio-functionality of these marine lipid bioactives, based on the paradigm of different cooking methodologies and thermal processing, while the compounds produced during such treatment(s) with detrimental changes in the fish lipid profile, which can reduce its cardio-protective efficacy, are also reviewed. Novel green extraction technologies and low temperature processing and cooking of fish and fishery by-products are needed to reduce these undesirable effects in a sustainable and environmentally friendly way.

## 1. Introduction

Seafood, and especially fish and its products, are currently acknowledged as sources of essential nutrients for humans with several health benefits [1], while current recommendations suggest the consumption of two servings of fish each week, with a minimum of one meal consisting of oily fish [2,3]. Consumption of fish and its products, including fish oils and food supplements based mainly on fish lipid bioactives, have increased significantly over the last few decades. The increase of the popularity for fish and such fish-derived products is mainly due to its excellent nutritional value, providing high quality nutrients [1,4], and especially highly bioactive lipid molecules, such as the omega-3 (n-3) polyunsaturated fatty acids (PUFA) [5], several bio-functional polar lipids (PL), marine carotenoids, and lipid vitamins, such as the vitamins A, E, and D [6,7].

Depending on their lipid content, fish can be classified into lean fish (<2.5% fat, such as cod and haddock), medium fatty fish (2.5–6% fat, for example seabass and hake), or fatty fish (>6–25% e.g., anchovy, salmon, sardine, mackerel, and herring). The lipid content, both quantitatively and qualitatively, vary depending on fish species, age, sex, and season [7]. Moreover, in contrast to other animal sources, fish have little contribution to the dietary cholesterol intake, with an average cholesterol content of 35 mg per 100 g of fish [7]. Fish are uniquely different from other animal sources also because of their lipid composition, containing up to 40% PUFA [1,6,8]. Oily fish such as salmon, mackerel, sardine, and tuna, their by-products, and their fish oils are the richest sources of the bioactive long-chain PUFA, such as the eicosapentaenoic acid (EPA; 20:5n3) and lower amounts of the docosahexaenoic acid (DHA; 20:6n3) [6,9].

The majority of worldwide fish oil production is mostly used in the aquaculture industry, while only a small proportion is used for the production of n-3 PUFA related products [10]. Thus, fishing of most species just for the production of fish oil is not a sensible or sustainable approach. Instead, reusing fish residues and side streams of processing, such as the head, liver, skin, trimes, etc., is considered a sustainable circular economy strategy, since fish by-products contain lipid ingredients and bioactive compounds, with high added value that could be used in the food and nutraceutical industries. Thus, the recovery of biologically valuable and desirable lipid compounds from marine fishery by-products, for the production of considerable amounts of bioactive fish oil and other valuable products, which can be employed for applications in human health and other industries (i.e., aquaculture, food, agrochemical, biotechnological, and pharmaceutical applications), has lately gained a lot of interest [10,11,12,13,14,15,16]. Hence, the production of lipid products containing fish lipid bioactives from marine by-products and side streams, can also help to reduce processing waste, thereby promote environmental protection, economic growth, and human health.

The health benefits of fish lipid products, either from fish or by valorizing fish by-products, have been mainly attributed to their rich content in n3 PUFA, such as DHA and EPA [10,11,12,13,14,15,16]. Consumption of fish oil rich in n3 PUFA such as DHA and EPA has been associated with several health benefits, such as improved platelet functionality and cardiovascular health [17,18], while a low value of the ratio of n6/n3 PUFA seems also to provide reduction of risk against CVD and other chronic disorders [19]. However, recent reviews and meta-analyses have highlighted that marine oil ω3 PUFA supplements such as purified fatty acids, esters, or moieties of triglycerides do not effectively affect the risk for chronic disorders as initially thought [17,20,21,22,23,24,25], while it has also been proposed in these studies that other beneficial lipid nutrients seem to contribute to the benefits of fish, fish oils, and fish lipid products [17,25].

Thus, apart from the neutral forms of PUFA, such as esters of PUFA and triglycerides containing PUFA, fish is also a good source of bio-functional marine polar lipids (PL) rich in n-3 PUFA, with potent antithrombotic, anti-inflammatory, cardio-protective, neuro-protective, and anti-tumor properties [6,9,26,27,28,29,30,31,32]. More specifically, bioactive fish PL, such as several phospholipids and glycolipids baring n-3 PUFA in their structure, possess much higher bioavailability of their n-3 PUFA content due to their amphiphilic properties. Consequently, fish PL rich in n-3 PUFA have been found to reduce the risk for inflammation-related chronic disorders such as CVD and improve neural function in much lower amounts, partly due to the higher bio-efficacy of their bio-functional n-3 PUFA [6]. Fish PL have also been suggested to have anti-inflammatory and anti-thrombotic properties and act as more effective modes of transport for n-3 PUFA than triglycerides to various organs of the body [6,33].

Fish also contain the important lipid-soluble vitamins A, E, and D, including natural antioxidants [34]. Lipid-soluble vitamins A and D generally emanate from the liver, and there is a notably high content of both found in the liver of codfish. However, a high content of both can also be found in the muscle mass of several fish species. The vitamin A content of fish fillet ranges between 3 and 180 ug/100 g. The vitamin D content of fish may vary as it is not correlated with the vat content and may have values ranging between 3–20 μg/100 g [7]. Apart from these lipid vitamins, Fish is known to contain several other vitamins from the B vitamin group including thiamine/vitamin B1 (40–210 μg/100 g), riboflavin/vitamin B2 (50–360 μg/100 g), niacin/vitamin B3 (2–10 mg/100 g), and pyridoxine (200–980 μg/100 g), which play an extremely important role in both metabolism and cell maintenance, as well as vitamin B12, also known as cobalamin (1–9 μg/100 g), which is imperative for DNA synthesis and helps maintain healthy blood and nerve cells [7].

Overall, a number of studies have also suggested that the favorable effect of fish intake on the cardiovascular risk is plausibly through the interplay of a variety of lipid nutrients found in fish and not just due to their high content of n-3 PUFA [17,25,35]. Therefore, it is now well established that it is the synergy of the various bio-functional lipid components and nutrients of fish and fish oils that exhibit beneficial effects on human health, rather than a simple fish lipid compound like the n-3 PUFA. The various lipid nutrients include the marine carotenoids and the lipid vitamins A, E, and D, but mainly several bio-functional PL, such as marine phospholipids and glycolipids rich in n-3 PUFA, and other functional fatty acids like the omega-9 (n-9) oleic acid (OA; 18:1n9).

Nevertheless, the quantity and quality of fish lipid bioactives, depend on many factors, including fish species, size and age, gender, diet, habitat temperature, season, and extraction methods and conditions, among others. The conditions and extraction method affect the composition and the quality of the lipids extracted from fish and fish processing side streams, and thus, selection of the method and its optimization are important considerations in producing fish lipid bioactives with desirable characteristics. One of the most detrimental parameters in fish processing associated with the reduction of fish lipid bioactives’ quality is thermal processing. Fish cooking, apart from its use for safe fish consumption, is also one of the first and crucial parts of the conventional methods of wet processing for the production of fish oils [36]. However, the highly unsaturated fish lipids that contain PUFA and monounsaturated fatty acids (MUFA) are susceptible to oxidation, especially under increased thermal procedures, as it is observed during several cooking processes, including those for producing fish oils. Fish muscle lipids contain large amounts of PUFA, which leaves them prone to oxidation. When thermal processing is applied to fish, it can cause the nutritional quality to be altered and inactivate enzymes and pathogens while increasing palatability. When looking at the results of popular cooking methods (e.g., steaming, boiling, and frying), these can cause undesirable physicochemical reactions, which mainly include lipid oxidation [37].

The resulting fish oil and products containing fish lipids derived from such conventional industrial processing that involves thermal treatment is on the one hand characterized by a high content of n-3 PUFA, while on the other hand several undesirable oxidation products and impurities are also present that comprise the rest of the oil component. Thus, in order to resolve the disadvantages associated with conventional methods and thermal processing, novel extraction techniques are being optimized to improve the quality and the oxidative stability of these high-value fish lipid bioactives, especially from sustainable fish sources, such as the valorization of fish by-products.

## 2. Fish Lipid Bioactives and Health Benefits

Fish is an important source of several essential nutrients and bioactive food compounds important for our health, such as the essential n-3 PUFA and other lipid bioactives, protein hydrolysates, polypeptides, peptides, amino acids, vitamins, carotenoids, and minerals [38]. Lipids are a class of extremely diverse biomolecules, holding a vast variety of functions and structures. Lipids are generally classified into two main subclasses depending on their polarity: the more neutral lipids (NL) and the more polar lipids (P). NL include mostly triacylglycerides (TAG), waxes, cholesterol esters, and long chain lipid esters and ethers, whereas PL include mostly glycolipids and phospholipids, while during some extraction procedures some lipid vitamins and marine carotenoids, such as the vitamins D, E, and A and astaxanthin, have been found to migrate to the PL fraction.

Fish have high content in bio-functional fatty acids, such as the essential n-3 PUFA alpha linolenic acid (ALA; 18:3n3), and the long chain n-3 PUFA, EPA and DHA, as well as bioactive MUFA, such as the n-9 MUFA OA. The majority of these fish PUFA and MUFA are esterified in carbon chains of neutral lipids, such as the TAG. In contrast, some amounts of these bioactive fish fatty acids are bound to the more polar PL, which are usually amphipathic/amphiphilic molecules, due to containing a polar hydrophilic group among with the hydrophobic hydrocarbon residue of these fatty acids [32]. The functional polar hydrophilic group is mainly either a carbohydrate-based or a phosphate-based polar head residue. These amphiphilic properties of marine PL provide new perspectives for their bio-functionality and bioavailability of their fatty acid content, as well as for their anti-inflammatory, anti-thrombotic, and cardio-protective potency [6,29,32].

It is now also well established that consumption of fish or fish products containing fish lipid bioactives, such as fish oil and supplements with long chain PUFA, have several health benefits, including reduced risk of CVD and coronary heart diseases, prevention in cardiac arrhythmias, as well as sudden death and the prevention of incidence of diabetes, among others (Table 1). The main health benefits of fish and fish oils were initially mostly accredited to their high n-3 PUFA content, particularly to the essential long chain ALA, EPA, and DHA, due to their anti-inflammatory effects on the eicosanoids’ related pathways [39]. It has been proposed that EPA and DHA in the form of fish oil supplements can decrease inflammation, platelet aggregation, heart rate, and blood pressure in humans [40] and consequently DHA and EPA (from fish) have been linked with a reduction in incidences of CVD, diabetes, cancer, and other inflammation-related diseases.

Subsequently, several recent meta-analyses have indicated inconclusive results to support the beneficial health effects of these neutral forms of n-3 PUFA, while it seems that several of these benefits are associated with a wide range of fish lipid bioactives with cardio-protective properties, rather than to the fish n-3 PUFA content only. Several of these lipid bioactives, individually and/or in synergism, have shown lowering of oxidative stress, inflammation, blood pressure, and improvement of vascular function and thus a reduction for the risk of atherosclerosis and CVD. Thus, an overall depiction of each class of lipid bioactives found in fish and especially the fatty acid content reported for commercially important fish species, along with an evaluation of their health benefits, is thoroughly described in this section.

### 2.1. Fatty Acid Content of Fish

Fish fatty acids are the class of lipids mostly researched in several fish species, and especially in oily ones, since as aforementioned, fish contain several bio-functional fatty acids with the essential PUFA being the most important ones. In Supplementary Table S1 we show the outcomes of several studies on the fatty acid composition and the SFA, MUFA and PUFA content, along with the n-6/n-3 PUFA ratios, of total lipids and PL from raw samples of important oily fish species, such as salmon, mackerel, seabass, seabream, and sardines, as well as that of cod—a lean fish used for comparison. As shown in Table S1, the fatty acid content varied between different fish species, and even within the same fish species, depending on whether the sample originated from wild catch or from cultured fish, or on the lipid sample analyzed for this fish species (for example, if the total lipids or the polar lipids were analyzed for the fatty acid content). Nevertheless, in all raw samples of both oily and lean fish species, the most prevalent from the PUFA were the n-3 PUFA ALA, EPA, and DHA and the omega-6 (n-6) PUFA linoleic acid (LA) (18:2n6), with favorably low values of the n-6/n-3 PUFA ratios, from the MUFA the most prevalent was the n-9 MUFA OA, and from the SFA was the palmitic and stearic acids.

#### 2.1.1. Saturated Fatty Acids (SFA) in Fish

SFA have been linked to increased blood levels of triglycerides (TG), total cholesterol (TC), and low-density lipoprotein (LDL) cholesterol. The cellular membranes and lipoproteins containing large amounts of SFA are less active functionally. Such lipoprotein particles form stable bonds with cellular receptors of lipoproteins, thus promoting disorders of the cholesterol transport system in the human body and leading to the development of the dyslipoproteinemias that contribute to atherosclerogenesis (atherogenic dyslipoproteinemia) [69]. Thus, SFA consumption is normally associated to severity of atherosclerotic lesions of the arteries and the development of CVD and other chronic diseases, while SFA should be decreased in foods and food products, including supplements, to avoid such metabolic diseases [70].

The fatty acid composition of fish is principally epitomized by a rather low content of SFA, which has also been proposed as being beneficial for fish or fish oil consumption versus other animal-derived protein and oil sources. SFA contained in fish are mainly C14:0 (myristic acid), C16:0 (palmitic acid), and C18:0 (stearic acid), which provides 9–50% of the total fatty acids. It was also reported that the fatty acid content and make-up of 34 marine water fish species ranged from 30.10% to 46.88% SFA, with palmitic acid and stearic acid being the primary acid SFA [71]. For the majority of the 34 marine water species in the studies, the contents of these saturated fatty acids were as follow: myristic acid (C14:0, 0.72–8.09%), palmitic acid (C16:0 15.97–31.04%), and stearic acid (C18:0 2.79–11.20%).

#### 2.1.2. Monounsaturated Fatty Acids (MUFA) in Fish

Throughout the past decades, MUFA have been recognized as being potentially beneficial for the reduction of CVD risk as well [72]. The majority of the studies on the benefits of MUFA were carried out mainly on the effects of oleic acid and palmitoleic acid on health. It remains inconclusive whether MUFA of chain lengths greater than 18 carbons have beneficial effects on chronic disorders. The early studies in Greenland Inuit Eskimo showed that high consumption of foods like fish that are rich in both long chain n-3 PUFA and MUFA possess cardioprotective properties, implying a potential correlation between long chain n-3 PUFA and MUFA intake and reduced risk of CVD [73], which is still debatable, since a lower risk of disease among the Inuit may be due to genetic differences in their diet.

In a study on the fatty acid composition of marine water fish species, the MUFA content of fish ranged from 11.83 to 38.17%, with palmitoleic acid and oleic acid being the most common [71]. The fatty acid composition of the most commercially sought-after European fish species, namely salmon, turbot, herring, and cod, contains significant amounts of MUFA. These fish contain approximately between 30 and 60% MUFA, in which erucic acid palmitoleic acid, oleic acid, and eicosenoic acid are the more prominent MUFA. The ranges of the MUFA content for the 34 marine water species in the study was as follows: palmitoleic acid (C16:1, 1.48–19.61%), and oleic Acid (C18:1 2.44–28.97%) [71]. Thus, oleic acid (C18:1) is the most prevalent MUFA in human foods, including fish. Oleic acid, independently of its origin-source, once ingested has been found to provide several health benefits, especially on the cardiovascular system [29,69], but also to have great potential against cancer, especially when combined with long chain PUFA from fish [74].

Oleic acid protects against cardiovascular insulin resistance, improves endothelial dysfunction in response to proinflammatory signals, and finally reduces proliferation and apoptosis in vascular smooth muscle cells that may contribute to an ameliorated atherosclerotic process and plaque stability [72]. Delgado et al. have also discovered a direct link of oleic acid with markers of inflammation and with heart failure [75], while oleic acid has also been found to possess strong anti-thrombotic potency by inhibiting platelet aggregation [76], Nevertheless, further research is needed on the impact of MUFA like oleic acid on CVD risk factors and clinical endpoints in order to elucidate a possible role of MUFA in primary and secondary prevention of CVD.

#### 2.1.3. Polyunsaturated Fatty Acids (PUFA) of Fish and the Importance of the n-6/n-3 PUFA Ratio

PUFA are fatty acids that contain two or more double bonds. The n-3 and n-6 PUFA are the most well known classes of PUFA, with the essential PUFA, n-3 ALA and n-6 LA, being the representative fatty acids of this class, respectively. The presence of a double bond at the 3rd or 6th carbon from the methyl terminus of the fatty acid chain signifies the nomenclature for the n-3 and n-6 fatty acids respectively. Both ALA and LA are essential fatty acids, meaning they cannot be produced by the human body. From these essential PUFA other long chain bio-functional PUFA can be produced, like the n-6 PUFA arachidonic acid (ARA) and the n-3 PUFA, EPA and DHA. However, humans are not only unable to synthesize ALA but also possess a limited ability to convert it to EPA or DHA, therefore the intake of these long chain n-3 PUFA from dietary sources, such as oily fish, is needed.

Increased levels of n-6 PUFA like LA and subsequently increased n-6/n-3 PUFA ratio promote the production of ARA and the pathogenesis of inflammation related diseases including CVD [19]. Inflammation is acknowledged as a factor of the pathophysiology of several chronic diseases such as CVD [29]. The commencement and undertaking of an inflammatory response involves the coordinated expression of many factors, such as cytokines, chemokines, growth factors and lipid mediators (eicosanoids), and platelet activating factor (PAF) [29,77]. Increased n-6 PUFA and ARA levels are also implicated in these inflammatory manifestations since it is a major substrate for eicosanoid synthesis promoted by these inflammatory mediators [19,29,77]. Eicosanoids are locally acting bioactive signaling lipids, which regulate the diverse set of homeostatic and inflammatory processes linked to numerous diseases [78].

The benefits of fish and fish oils have long been accredited to their high n-3 PUFA content and mainly to their antithrombotic and anti-inflammatory properties. n-3 PUFA exert both anti-atherogenic and anti-thrombotic effects [79]. It has been proposed that fish n-3 PUFA act mainly as precursors to several anti-inflammatory compounds that decrease the formation and tissue incorporation of the n-6 PUFA ARA and its inflammatory eicosanoid products. More specifically, the prostaglandins and leukotrienes that are established from EPA by cyclooxygenases (COXs) and lipoxygenases (LOXs) are less pro-inflammatory than those derived from ARA [80].

Since n-3 PUFA induce the increase of anti-inflammatory eicosanoids that act as antagonistic inhibitors to the inflammatory n-6 PUFA derived eicosanoids, the ratio of n-6/n-3 PUFA in the diet can be extremely important [19]. The ratio for n-6/n-3 PUFA in the westernized diet has risen drastically in comparison to the 1:1 ratio of our ancestors’ diet. These drastic changes coincide with the increase of inflammation, obesity, and related disorders. In 2002, the ratio of the western diet ranged from 15/1 to 16.7/1 and is now approximately 20:1. Western diets have a low intake of n-3 PUFA and have excessive levels of n-6 PUFA in comparison to the diet which humans evolved from and which their genetic patterns were established from [19]. Therefore, the lower the n-6/n-3 PUFA ratio in a food such as fish or in an overall diet, the better the health outcome against inflammation and thrombosis related chronic disorders [19].

Nevertheless, several other mechanisms have also been proposed that are not limited to these effects of n-3 PUFA on the eicosanoids’ pathways, which also include among others the effects of n-3 PUFA on inflammation, beta oxidation, endothelial dysfunction, cytokine growth factors, and gene expression of adhesion molecules, without however an adequate explanation of the beneficial actions and of n-3 fatty acids [81]. For example, the n-3 PUFA are known to inhibit the actions of the pro-inflammatory transcription factor nuclear factor κB (NF κB), which instigates the expression of many pro-inflammatory genes that encode adhesion molecules, chemokines, cytokines, and various other effectors of the innate immune response [82]. The close interaction between the central nervous system, endocrine organs, cytokines, exercise, and dietary n-3 fatty acids is also associated with the cardioprotective and neuroprotective action(s) of n-3 fatty acids, which have been found to suppress TNFα and IL synthesis and release, to modulate hypothalamic-pituitary-adrenal anti-inflammatory responses, and to increase in acetylcholine release, the vagal neurotransmitter [81].

Thus, the high n-3 PUFA content in fish and their anti-inflammatory properties seem to contribute greatly to the nutritional health benefits of fish and fish oil. Subsequently, PUFA of marine origin, particularly the long chain n-3 PUFA such as EPA and DHA, have been investigated for their effects against several inflammation related diseases including CVD, cancer, Alzheimer’s disease, diabetes, and a number of central nervous system disorders [81,83], while PUFA especially DHA is of benefit to both the brain and visual systems, as well as reducing heart problems [81,84]. Recent research has also postulated that consumption of EPA and DHA may also affect the functions of the immune system and the reproduction system.

Since the ‘90s, fish and n-3 PUFA intake have been linked to a reduced risk of coronary heart disease (CHD) [85,86,87]. Soon after, n-3 PUFA intake was found to lower cholesterol levels, low density cholesterol (LDL) levels, and triglycerides, while also slightly increasing high density cholesterol (HDL) levels [79]. A quantitative analysis on fish consumption described that individuals who consumed fish had a 17% decreased risk of CHD mortality, in comparison to no fish consumption [54]. Their hypothesis was also supported, stating that n-3 PUFA intake reduces the tendency for arrhythmias development. Further results showed that an incremental increase of fish intake by one serving per week led to a further decrease of 3.9% in CHD risk, while also getting enough evidence to support their second hypothesis that n-3 PUFA intake reduce the development of atherosclerosis. The results of this study were also in agreement with the findings of [55]. The results indicated that fish consumption was inversely associated with CHD risk. More importantly, it was reported that a 7% decreased risk of CHD mortality was associated for every 20 g/day of fish [55], in comparison to 5.5% reduction in the study carried out by [54] (Table 1).

Epidemiological studies have shown great evidence that fish lipids and oils favorably affect CHD mortality (Table 1). The Chicago Western Electric Study reported that men who consumed >35 g/d of fish had a reduced relative risk of death from CHD of 0.62 and a relative risk of non-sudden death from myocardial infraction (MI) by 0.33, in comparison to men who did not consume fish [88]. The results of an ecological study showed that fish consumption was associated with a reduced risk for all causes of heart disease and stroke mortality [89]. In addition, a Japanese study reported a dose-response relationship between fish consumption and the reduction of CVD risk factors [90]. The Nurses’ Health Study reported an inverse association between fish consumption and CHD death in women [91]. The findings showed that the reduced risk of CHD was 21% for individuals who consumed fish few times per month, 29% in individuals who consumed fish once per week, 31% in those who consumed fish several times per week, and 34% in those who consumed fish more than 5 times per week. In the Physicians’ Health Study, consumption of fish at least once per week was found to have a 0.48 relative risk of sudden death in comparison to individuals who consumed it less than once per month [92]. There is a multitude of evidence supporting that fish consumption, especially for n-3 PUFA, has a reducing effect on the risk of coronary heart disease.

Fish consumption has also been proposed by several epidemiological studies to reduce the risk of stroke as well [79]. However, several researchers have initially failed to find an association between the two [79]. Nevertheless, a meta-analysis comprised of prospective studies reviewed 33 studies on the association between fish consumption and the risk of stroke. It was found that a higher intake of fish was associated with reduced risk of stoke as a clear distinctive relationship between fish consumption and stroke was detected [93].

There is a multitude of evidence that suggests the possibility that omega-3 fatty acids could potentially be able to reduce the risk of sudden death [92,94,95]. Consuming fish at least once per week has been associated with an increase in heart rate variability in MI survivors [96]. An intake of n-3 PUFA, EPA, and DHA, has shown to reduce the resting heart rate as well as increase the left ventricular filing capacity [59]. Animal experiments have demonstrated the potent antiarrhythmic effects of fish oil. Studies conducted with animals have found an association between n-3 fatty acids and a reduction in damage done to the cardiac muscle and a forestalled development of ventricular dysrhythmias [97]. In addition to this, cats that were supplemented with fish oil were protected from cerebral damage after stroke induction [98]. n-3 PUFA are powerful inhibitors of sodium channels in neonatal cardiac myocytes, contributing to the reduction in arrhythmia [99]. A cross-sectional analysis observed whether more frequent fish consumption was associated with lower rheumatoid arthritis. Food frequency questionnaires were used to evaluate the diets of the 176 participants. The findings showed that increased consumption of fish may be associated with lower disease activity in RA patients [58].

A low incidence rate of diabetes mellitus, one of the major risk factors for CVD, has been associated with fish consumption [79]. n-3 PUFA have shown to improve numerous metabolic factors of insulin resistance by the lowering of hypertension and circulating plasma triglyceride levels [73]. In a meta-analysis, a significant reduction in blood pressure of −3.4/−2.0 mm Hg was found in individuals who have underlying hypertension who consumed 5.6 g/d of omega-3 fatty acids [100]. Likewise, blood pressure was reduced by −5.5/−3.5 mm Hg in individuals with untreated hypertension who were given >3 g/d of omega-3 fatty acids [100]. In view with the high doses required to lower blood pressure, an increased intake of omega-3 fatty acids has been found to have a minimal role in the management of hypertension.

Several randomized clinical trials (RCTs) have also been employed to investigate the benefits from fish consumption and of its n-3 PUFA content (Table 1). One of the first RCTs to investigate the cardioprotective effects of fish was the “Diet And Reinfarction Trial”, which reported a 29% reduction in all-cause mortality in male MI survivors who were instructed to increase their consumption of oily fish [43]. The most-notable benefit was seen in the decrease of fatal MI, and the protective effects were attributed to the *n* = 3 PUFA content of fish. Patients who were admitted to the hospital due to suspected MIS were randomized to consume fish oil, mustard oil, or a placebo. The results of this RCT showed that total cardiac events were 25%, 28%, and 35% in individuals randomized to intake fish oil, mustard oil, and a placebo, respectively [44]. A significantly lower risk of non-fatal MIs was noted in the groups randomized to intake fish oil and mustard oil.

One of the largest prospective RCTs that tested the beneficial effects of omega-3 fatty acids for the secondary prevention of CHD was the GISSI-Prevention Study [45]. A total of 11,324 patients with pre-existing CHD were randomized to take 850 mg of omega-3 fatty acid ethyl esters, 300 mg of vitamin E, both, or neither. After a 3.5-year follow-up, the group randomized to take omega-3 fatty acids alone experienced a 15% reduction in the primary end point of death, nonfatal stroke, and nonfatal MI. The incident rate of all-cause mortality was reduced by 20% and sudden death was reduced by 45%, in comparison to the vitamin E group, which reported no additional benefits. There was a 4% and a 2.5% reduction in triglyceride and LDL-cholesterol levels, respectively. A trial that had practical intakes of omega-3 fatty acids had individuals randomized to consume omega-3 or a placebo [46]. The results reported a significant lower progression, more regression, and fewer clinical events in the subjects randomized to consume omega-3 fatty acids. A meta-analysis of 11 RCTs concluded that the risk ratio of nonfatal MI, fatal MI, and sudden death was 0.8, 0.7, and 0.7, respectively [47].

A more recent RCT studied the efficacy of a Mediterranean diet supplemented with high n-3 fatty fish intake in Greek asthmatic children. Sixty-four children successfully completed the trial and after adjustment of age, sex, BMI, and regular physical activity, it was concluded that two fatty fish meals per week could potentially reduce airway inflammation in childhood asthma [56]. The HEL-FIMED study was one of the first RCTs to show how dietary changes, supplemented with fish oil, can improve mental health in people with depression. This study randomized individuals to a Mediterranean diet style dietary pattern, supplemented with fish oil. After 3 months, reduced depression was associated with an increase in the Mediterranean diet score, and after 6 months mental health was sustained [57].

The inverse association of fish consumption or intake of fish n-3 PUFA with various inflammatory markers is also well documented [48,49,50]. There was also a strong inverse association of n-3 PUFA and inflammatory cytokines in the Health 2000 survey, particularly regarding TNF-a and IL-6 [51]. One of the most notable studies that examined the effect that fish consumption had on the levels of inflammatory markers in relation to CVD was the ATTICA study. This cross-sectional study consisted of 1514 men and 1528 women who had no previous clinical evidence of any cardiovascular issues. All inflammatory markers such as C-reactive protein (CRP), IL-6, TNF-a, serum amyloid A (SAA), and white blood cells (WBC) showed a strong inverse dose response relationship with the intake of fish [52]. The most significant differences were observed when high fish consumption was compared to no consumption. Individuals who consumed more than 300 g of fish per week, had on average 33% lower CRP, 33% lower IL-6, 21% lower TNF-a, 28% lower SAA, and a 4% lower count of WBC [52]. Furthermore, meta-analysis comprised of observational studies observed that individuals who consumed fish had a 15% reduced risk of coronary heart disease mortality, compared to individuals who did not consume fish [101]. The consumption of fish oil had been suggested to decrease CRP and IL-6 circulating levels in postmenopausal women [102]. In addition, a decrease in TNF-a and IL-6 levels was observed in peripheral blood mononuclear cell production with an increasing intake of n-3 PUFA [103].

A meta-analysis that investigated the effect of a diet supplemented with fish oil on inflammatory markers in relation to coronary heart disease, concluded that fish oil supplementation has a suppressive effect on circulating TNF-a, IL-6, and IL-1, suggesting that fish oil supplementation can help prevent an inflammatory response and reduce chronic heart failure (CHF) [53]. The results concluded that a higher dose or a longer follow-up period were associated with a more remarkable reduction in TNF-a and IL-6. In the studies that included meta-analysis of IL-1, the dosage of fish oil was >1000 mg/day and the follow-up periods were 6–12 months. There is a clear inverse association between fish intake and levels of pro-inflammatory markers. Therefore, there is sufficient evidence to say that fish consumption suppresses inflammation and provides beneficial effects on human health [52]. Thus, a direct dietary intake of foods rich in the semi-essential EPA and DHA, such as fish and fish oil, is necessary, since consuming 1–2 servings of fish weekly, especially fish high in EPA and DHA, correlates to a reduction in the risk of both coronary death (36%) and total mortality (17%), and an intake of 250 mg of EPA and DHA daily was deemed as sufficient for primary prevention [104].

In general, marine organisms have been recognized as the sole foods that contain naturally high concentrations of EPA and DHA, which they have accumulated through the phytoplankton in their food chain. The levels of EPA and DHA however varies amongst species dependent on the lipid content of the fish. Oily fish such as salmon, sardines, herrings, and mackerel tend to have high levels of such long chain n-3 PUFA, in comparison to some leaner fish like cod and halibut.

Overall, an intake of at least 0.25 g/day of fish derived n-3 PUFA is recommended by European food safety authority (EFSA) and World health organization (WHO). A moderate consumption of fish, as it is applied in healthy diets, such as the Mediterranean diet, can help to achieve this recommended intake. However, a lot of people do not choose to include fish as part of their weekly diet for several reasons, such as adopting a more vegetarian/vegan-based type of diets. Thus, due to the benefits of fish lipids, and in order to cover the nutritional needs of all these people, products such as food supplements and fish oils rich in n-3 PUFA have gained a big area in the food supplements’ market. The supplementation of the n-3 PUFA and mainly of esters of EPA and DHA, have been well documented in scientific literature for both their anti-inflammatory and hypolipidemic properties. However, according to EFSA, high amounts of neutral forms of n-3 PUFA bound to esters or TAG are needed in supplements, in order to provide any anti-inflammatory or cardiovascular benefit. More specifically, EFSA have recommended 2–4 g/day of these neutral forms for any of these health benefits to take place, which are much higher than the ones achieved by moderate fish consumption in a healthy diet (0.25 g/day) [67].

Subsequently, recent randomized control trials and systematic reviews and meta-analyses studies have had inconclusive results for the beneficial health effects of n-3 PUFA supplementation [6]. For example, the Alpha Omega Trial in 4837 post-myocardial infarction patients was a 2-by-2 factorial design with 2 g ALA or 400 mg EPA/DHA as the interventions, and no significant effect was found for either EPA/DHA or ALA [64]. Most importantly, a very recent randomized, placebo-controlled trial, VITAL trial (Vitamin D and Omega-3 Trial), showed that supplementation with n-3 PUFA did not result in a lower incidence of major cardiovascular events or cancer than the placebo [20].

Systematic reviews and meta-analyses have also recently highlighted that n-3 PUFA in form of purified fatty acids or esters, did not exhibit benefits in at risk patients and does not affect the risk of all-cause death [6]. A characteristic example is a specific recent meta-analysis study and systematic review of such randomized control trials on the relationship between n-3 PUFA supplementation and risk and occurrence of major CVD events, where it was highlighted that there is a lack of evidence to suggest the beneficial effect of n-3 PUFA supplementation in respect to cardiovascular events and other measurable changes in health [21], while similar outcomes were also observed in adults with peripheral artery disease (PAD) [22]. In both these studies, n-3 PUFA supplementation did not decrease the threat of all-cause mortality, cardiac death, myocardial infarction, or stroke based on relative and absolute measures of association.

Moreover, another meta-analysis study also looked into the efficacy of EPA and DHA supplementation for the secondary prevention of CVD, and noted a small reduction in cardiovascular death. However, a study with extensive methodology issues was removed this reduction disappeared, concluding that there is no adequate evidence of a secondary preventive effect of n-3 PUFA supplements against cardiovascular events in patients with a history of cardiovascular disease [24]. Additionally, in a systematic review of placebo-controlled randomized controlled trials (RCTs) of n-3 PUFA supplementation, which enrolled >1000 patients with a follow-up of more than a year and meta-analysis of RCTs, an insufficient evidence supporting the routine use of n-3 PUFA in both primary and secondary prevention of CVDs was established [23]. It was also suggested by authors that pharmacists are ideally situated to discuss the lack of benefit and possible risk of n-3 PUFA supplements with patients. n-3 PUFAs may interact with medications that affect homeostasis (anti-platelet agents- warfarin) and may increase risk of bleeding [23]. Related outcomes were also obtained in another systematic review and meta-analysis on the relationship between fish consumption (long chain n-3 PUFA) and risk of cerebrovascular disease [25].

The Prevention of Post-operative Atrial Fibrillation (OPERA) study involved a randomized control trial on the n-3 PUFA to determine whether peri-operative oral n-3 PUFA reduces the occurrence of post-operative atrial fibrillation in 1516 patients receiving cardiac surgery [65]. The treatment comprised of the administration of 8–10 g of n-3 PUFA or placebo divided over 2–5 days followed by 2 g per day until discharged from hospital or post-operative day 10—whichever event occurred first. Results concluded that n-3 fatty acid administration did not reduce the risk of post-operative atrial fibrillation relative to the placebo [65]. The ORIGIN (Outcome Reduction with an Initial Glargine Intervention) trial examined the hypothesis that long-term supplementation with n-3 fatty acids in the form of 1 g capsules containing at least 900 mg of ethyl esters of n-3 fatty acids would reduce the rate of cardiovascular events in patients with either Type II diabetes, impaired fasting glucose, or impaired glucose intolerance [66]. The trial consisted of 12,536 participants during an average follow-up of 6.2 years and concluded that incidences of death from cardiovascular causes did not decrease significantly amongst patients that received n-3 fatty acids, as compared to the patients that received the placebo [65].

On the one hand it has been suggested that a high dietary intake of both marine and nonmarine-based n-3 PUFA is associated with a reduced risk of cardiovascular death in the Chinese population, particularly for deaths from coronary heart disease and in individuals without cardiovascular disease at baseline [35], while it has also been suggested by several other researchers that the favorable effect of fish intake on cerebrovascular risk is likely to be brought about through the interplay of a number of nutrients abundant in fish [25]. Apart from n-3 PUFA in the form of esters, various other fish lipid bioactive nutrients, such as the lipid vitamins A, D, and E, fish carotenoids and mainly the fish polar lipids (phospholipids and glycolipids) that are rich in n-3 PUFA, contribute greatly to the anti-inflammatory, anti-thrombotic and antioxidant cardio protective properties of fish lipids and the reduction of CVD risk [6,29,32].

Nevertheless, unsaturated fatty acids, including the n-3 PUFA, are unfortunately prone to oxidation. Oxidized formation of unwanted by-products can occur, such as hydroperoxides and aldehydes [42], which are highly toxic compounds, some of which can possess PAF-like structures and inflammatory activities [28,29]. These PAF-like molecules can cause oxidative stress by mimicking the inflammatory and thrombotic activities of PAF, which causes platelet aggregation and blood vessel dilation and in turn may lead to thrombosis, as well as to several other thrombo-inflammatory manifestations implicated in endothelial dysfunction, atherosclerosis, CVD, and several tumors [28,29]. Therefore, an accumulation of these oxidized compounds can cause oxidative stress and trigger an inflammatory response [29].

For this reason, appropriate measures should be applied when obtaining fish lipid bioactives, including EPA and DHA, in order to avoid the possibility of their oxidation and the production of unwanted by-products. Green technologies and as low as possible temperatures to be applied during the “cooking” techniques for obtaining fish lipids are needed to reduce the risk of fish lipids’ oxidation. Recent studies suggest also that the most effective way to prevent this oxidation involves the binding of n-3 PUFA into the structures of PL, which are less prone to oxidation than the neutral forms of n-3 PUFA. In addition, the presence of natural antioxidant compounds in several fish and fish oils, such as free-radical scavengers, active-oxygen scavengers, metal binders, peroxide destroyers, antioxidant enzymes, polar lipids with antioxidant stability, lipid vitamins and various marine carotenoids like astaxanthin, play a crucial role in protecting against oxidation, fish MUFA, and PUFA [105]. Apart from these antioxidant activities, several other health benefits have also been proposed for both fish lipid vitamins and fish carotenoids like astaxanthin [106], which further suggests the favorable and needed co-presence of fish PL, lipid vitamins, and carotenoids in an extract containing fish lipid bioactives.

### 2.2. Fish Polar Lipids (Phospholipids and Glycolipids)

The importance of n-3 PUFAs at cellular levels for maintaining membrane homeostasis, its influence on gene expression, and its vital importance for an optimal balance with n-6 PUFA to regulate the inflammatory response have been demonstrated in numerous studies. The majority of these clinical studies were carried out using n-3 PUFA bound TAG or ethyl esters. Recently, the importance of fish products containing n-3 PUFAs bound to PL has come to prominence and the health benefits have been clinically observed.

**Figure 1:**
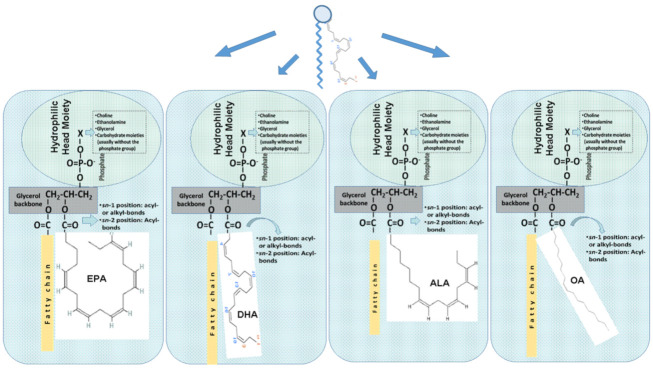
Structures of representative fish PL bioactives. Abbreviations: EPA = eicosapentaenoic acid (C20:5n3); DHA = docosaexaenoic acid (C22:6n3); ALA = alpha linolenic acid (C18:3n3); OA = oleic acid (C18:1n9).

When present in nature, n-3 PUFA are generally esterified either to PL or TAG or are present in the free form due to partial hydrolysis. PL generally have two fatty acids esterified to a glycerol- or a sphingosine-based backbone, and a phosphorus functional group for phospholipids or a sugar for glycolipids that is linked to a head group (Figure 1). TAG, consisting of three esterified fatty acids to a glycerol backbone, are highly hydrophobic, whereas PL are amphiphilic molecules since they have both hydrophilic properties due to their polar head group and hydrophobic properties due to their fatty acid chains. Due to this difference in hydrophobicity, only PL can form micelles and liposomes, while the biological importance of these PL is derived mostly from their amphiphilic properties. Fish and marine foods contain less amounts of bioactive PL, either glycolipids or phospholipids, and especially those baring n-3 PUFA in their structures, than TAG. Even though having smaller quantities than TAG, these dietary marine PL exhibit potent anti-inflammatory and anti-thrombotic properties with a plethora of health benefits [6,9,26,27,28,29,30,31,32,63].

In general, oily fish, namely mackerel, salmon, and herring, are the leading sources of marine PL. The classes of important fish phospholipids are mainly glycerol-based phospholipids, with some amounts of sphingo-based phospholipids and ether glycerol-based phospholipids. Glycerol-based phospholipids can be classified into different subgroups defined by their polar head group (Figure 1). The head group can comprise of choline, ethanolamine, serine, glycerol, or inositol. The most plentiful PL present in oily fish are phospholipid molecules of phosphatidylcholine (PC), followed by phosphatidylethanolamine (PE), phosphatidylinositol (PI), phosphatidylserine (PS), lysophosphatidylcholine (lyso-PC), and sphingomyelin, which are also present, but in lesser quantities. The principal PL class of oily fish-derived PL is usually PC, typically rich in n-3 PUFA at their sn-2 position of their glycerol backbone, predominantly EPA and DHA (Figure 1) [9,30,31]. Thus, the most prevalent n-3 PUFAs in fish PL are EPA and DHA, and to a lesser extent docosapentaenoic acid (DPA) and stearidonic acid [68]. Depending on the species, the fish may contain up to one third EPA and DHA in the form of PL.

Other PL also include the glycolipids, which can be either glycerol-based glycolipids or sphingo-based glycolipids [107]. Glycolipids represent a wide range of natural PL consisting of a glycosidic fragment attached to a lipid molecule. Glycerol-based glycolipids are molecules based in glycerol as the backbone with two fatty acids esterified in the 2nd and 3rd position of this backbone, while there is a monosaccharide/oligosaccharide molecule (polar group) at the 1st position of the glycerol (Figure 1). The sphingo-based glycolipids are a more complex subclass of PL, with several types of sphingosine-bases and a fatty acid being esterified in one of the positions of this backbone, while in the 1st position there are several monosaccharide/oligosaccharide polar functional groups bound to the sphingosine base. Several glycolipids also carry a functional sulphate group bound in the sugar group, also found in marine sources and especially in microalgae, and thus through the food chain to fish as well [108,109].

Glycolipids have an important role in conferring certain biological, physical, and chemical properties to carrier molecules, therefore making them crucial for cellular-recognition and cell-cell interaction processes [107]. Glycolipids of marine origin represent an important class of natural products with broad structural diversity and a wide range of biological activities, including antibiotic, antitumoral, antimalarial, antiviral, immunostimulatory, and neurogenic activities [107]. In addition, strong anti-inflammatory and antithrombotic properties have been attributed to several glycolipids from fish [26] and microalgae, including to those with a sulpho-group [108,109].

All dietary marine PL are digested and absorbed in different ways in the small intestine than TAG. In contrast to TAG digestion, which requires their emulsification by bile salts, PL are not hydrolyzed by lingual or gastric lipases but only in the small intestine. Thus, after dietary intake, PL are almost completely absorbed in the intestine [32]. Most PL are hydrolyzed at the sn-2 position by the pancreatic phospholipase A2 (PLA_2_) in the lumen, and then absorbed by the enterocytes as free fatty acids (FFA) and lyso-PL, which are again re-esterified to PL (while some FFA are incorporated to TAG) and enter the bloodstream incorporated in the surface layer of chylomicrons (in contrast to TAG, which are incorporated into the cores of chylomicrons) but also in a small proportion in Very Low Density Lipoproteins (VLDL). After degradation to the TAG-rich particles of chylomicron, PL and their intact fatty acids can be taken up by HDL, which occurs relatively rapidly, that is, within 5–6 h of PL ingestion. From HDL, PL and their fatty acid content can be transferred into cells of numerous tissues and organs [68].

Interestingly, almost 20% of intestinal marine PL are absorbed passively and without hydrolysation, and preferentially incorporated directly into HDL [68]. In addition, a substantial part of the dietary PL fraction is integrated into HDL-particles already in the intestine that later join the plasma HDL pool. There is also some evidence that incorporated PL in the lipoproteins of the blood stream, might be a more efficient delivery form than TAG for PUFA to several tissues and organs (i.e., brain, liver, lung, heart, etc.) and even in blood cells such as platelets and erythrocytes [32,68]. Thus, dietary PUFA bound in PL differently affect the composition of HDL and LDL, especially the surface of these lipoproteins and subsequently their interactions with cells, in contrast to PUFA bound in TAG that are usually accumulated in the core of these lipoproteins. Subsequently, marine PL rich in PUFA differently affect the composition and functionality of lipoproteins and their distribution in the body and fatty acid tissue incorporation. Dietary marine PL are incorporated preferably into the surface of HDL and thus directly affect the levels and functionality of this “good” cholesterol lipoprotein, which remove excess cholesterol from blood stream and from atherosclerotic plaques and also possess strong anti-inflammatory and antioxidative properties, contributing to the reduction of LDL oxidation, and subsequently to the reduction of PAF produced by such oxidation and plasma oxidised PAF-like lipids, concluding in the maintenance of endothelial cell homeostasis which protect the cardiovascular system [6,32].

Thus, bioactive fish PL, like phospholipids and glycolipids baring n-3 PUFA in their structure, possess much higher bioavailability of their n-3 PUFA when compared to neutral forms of TAG or lipid esters of the n-3 PUFA, due to their amphiphilic properties (they “travel” in plasma lipoproteins and are incorporated into cell-membranes more easily, including surpassing the blood-brain barrier) [6,29,32,68]. Consequently, fish PL rich in n-3 PUFA have been found to reduce the risk for inflammation-related chronic disorders, such as atherosclerosis and CVD, and improve neural function in much lower amounts, like those found in moderate consumption of fish (0.25 g/day), partly due to their higher bioavailability of their bio-functional n-3 PUFA content [6,9,26,27,28,29,30,31,32,60,63]. Fish PL have been suggested to act as more effective modes of transports for n-3 PUFA [33,68], and thus having higher anti-thrombotic and anti-inflammatory bio-efficacy [9,26,27,30,31,60]. Moreover, unlike n-3 PUFA, marine PL are less prone to oxidation [32,105]. This may be on account of the natural presence of antioxidants of polar nature within the PL formation in cells and foods, such as the potent antioxidant astaxanthin found in salmon and microalgae or the lipid vitamins A, E and D.

PC derived from fish eggs (i.e., salmon roe) can also reduce chronic liver disease, as it was shown in six months of such an administration in a small human trial [110]. Results showed a decreased serum globulin and a rise in apolipoprotein A–I and E levels. Results also showed an increase in HDL. Nevertheless, no other blood parameters of importance for liver function were affected [110]. Moreover, in a six-week human study it was showed that marine PL from salmon roe may aid in the prevention of tumor-associated weight loss [111]. On a daily basis, the patients received 1.5 g marine PL over the course of six weeks and body weight stabilization was achieved in addition to an improvement in both appetite and quality of life [111].

**Figure 2:**
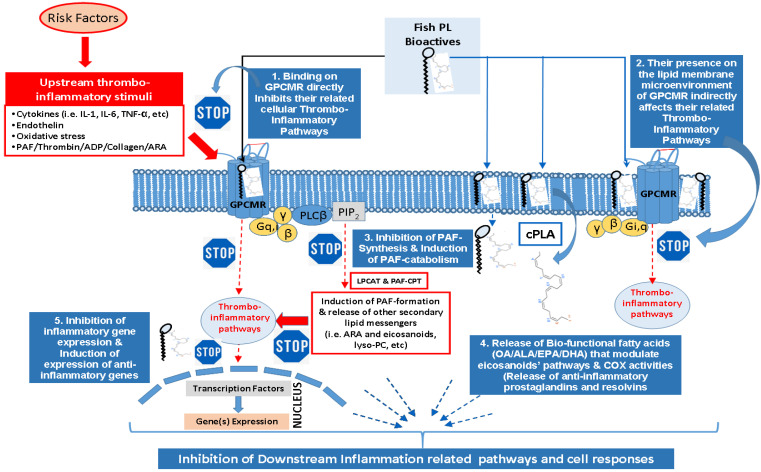
Modes of beneficial actions of fish PL Bioactives (in blue color) against the thrombo-inflammatory related pathways and cell-responses (red color). (Abbreviations: PAF = platelet-activating factor; ADP = adenosine 5′ diphosphate; GPCMR = G-protein coupled membrane receptors; cPLA_2_ = cytoplasmic Phospholipase A_2_; LPCAT & PAF-CPT = Basic regulatory biosynthetic enzymes of PAF; ARA = arachidonic acid; COX = cycloxygenases).

However, it should be stressed that PL by themselves have exhibited several beneficial effects, without and unrelated to the added benefits of their n-3 PUFA content. Some studies have shown that PL may help to alleviate senescence and benefit cognitive function [112,113,114]. Most importantly, fish PL have also modulated or even reduced the formation of arteriosclerotic plaques [61], by reducing the levels of the inflammatory and thrombotic mediator, PAF, and its atherogenic effects (Figure 2) [26,27]. Apart from modulating PAF-metabolism towards reduced PAF-levels, fish PL have also inhibited the inflammatory and thrombotic pathways of both PAF and Thrombin, while they have reduced the platelet activation and aggregation induced by well-established platelet agonists, collagen, and ADP [9,30,31,60,63], and also showed strong anti-inflammatory activities in general (Figure 2) [115], with several proposed health benefits [28,29]. Marine polar lipid bio-functional compounds such as fish PL have been suggested to have higher anti-inflammatory and anti-thrombotic bio-efficacy [6,28,32], and act as more effective modes of transport for PUFA than triglycerides to various organs of the body [33,68].

For example, sea bass and sea bream contain bioactive PL with potent beneficial bio-functionality against the platelet aggregation induced by the highly bioactive inflammatory and thrombotic mediator PAF [63], while they were also found to inhibit the enzymatic activities of the regulatory enzymes of PAF-biosynthesis (Figure 2) [27]. These bioactive sea bream PL have also been found to have anti-atherogenic properties since they reduce the formation of arteriosclerotic plaque [61] and putative antitumor properties, by increasing the levels and functionality of HDL cholesterol and by reducing PAF-levels and thus the inflammatory and thrombotic processes induced by PAF (Figure 2) [26,27,28]. The latent anti-PAF properties of fish PL usually occur either through affecting beneficially PAF-metabolism towards reduction of its levels of homeostatic ones and/or through inhibiting the binding of PAF on its receptor and thus inhibiting PAF-related inflammatory and thrombotic pathways and activities (Figure 2), and subsequently reducing the risk for inflammation related chronic disorders such as atherosclerosis, CVD, and cancer [26,27,28,29].

Apart from the strong anti-inflammatory and anti-thrombotic properties of fish PL, PL from oily fish rich in n-3 PUFA, such as salmon and herring, showed also very low levels of their n-6/n-3 PUFA ratio [9,30,31,60], which further supports the higher anti-inflammatory bio functionality and cardio-protective properties of fish PL, since the lower the values for this ratio, the higher the protection against inflammation and related chronic disorders, including CVD [19]. The rich in n-3 PUFA PL are usually transferred from plasma lipoproteins to cell-membranes, where a cytoplasmic PLA2 release the n-3 PUFA from the *sn*-2 position of these membrane bound PL, while the released n-3 PUFA interacts with the eicosanoids pathways (COX-enzymes) for reducing and resolving inflammation and the inflammatory cell-response (Figure 2). Similar PL bioactives have also been found in another oily fish—mackerel—with potent anti-thrombotic and anti-inflammatory properties against the pathways of both PAF and thrombin [116]. The PL of mackerel were found to have more potent anti-PAF and anti-thrombin properties than the neutral lipids, and thus were proposed for stronger anti-atherogenic properties too [116]. Another oily fish—sardines—have also been found to contain bioactive PL rich in n-3 PUFA, possessing strong anti-inflammatory activity against PAF action [117]. The cardioprotective properties of raw sardines has also been proposed to be related to the high n-3 PUFA content found in raw sardines, while the n-6/n-3 PUFA ratio was favorably very low, approximately 0.1 [117], which further supports the anti-inflammatory and cardioprotective properties of sardine lipid bioactives too.

In comparison to all the aforementioned oily fish of commercial interest, another commercially important fish is cod, which has been characterized as a lean white fish, due to its less overall lipid content. Nevertheless, it has also been found that cod possess bio-functional lipids too, with the PL fraction exhibiting the most prominent anti-platelet properties against platelet aggregation induced by the inflammatory and atherogenic mediator PAF and the thrombotic inducer thrombin, which further suggest the anti-atherogenic and cardio protective properties of PL from raw cod [118].

Thus, independently of the fish lipid content, fish PL from several fish species showed strong bioefficacy against inflammation and thrombosis. There is substantial evidence for the potent antithrombotic, anti-inflammatory, cardio-protective, neuro-protective, and anti-tumor properties of bioactive fish PL against relative inflammation-associated chronic disorders, such as atherosclerosis and CVD, renal and neurodegenerative disorders, autoimmune diseases like rheumatoid arthritis and Lupus, cancer and tumor metastatic procedures, as well as persistent infections [6,9,26,27,28,29,30,31,32,60,61,62,63,68,110,111,112,113,114,115,116,117,118]. Recent reviews have extensively looked at several studies and clinical trials based on the bio-efficacy of fish PL and subsequent health benefits [6,27,28,32,68]. Nevertheless, more in vivo studies and targeted randomized control trials are needed to fully evaluate the health benefits of fish PL.

### 2.3. Fish Alkylacylglycerols

Alkylacylglycerols (AKG) are accumulated in the liver of certain marine fish species such as chimeras. They are mainly bioactive ether lipids that are especially abundant in oil extracted from the liver of chimeras and sharks, where they account for up to 50% of the liver fraction [119]. Bioactive compounds present in shark liver oil, namely AKG and its derivatives, have been demonstrated to influence various physiological mechanisms in the human body. AKG have previously been reported to play an imperative role in the modulation of immunity through the enhancement of macrophage activation and increasing the plasma levels of immunoglobulin in rodents. In vitro studies have suggested that AKG play the role of antimicrobial agents and exhibit various biological activities and great therapeutic potential [120].

AKG participate in the regulation of key biochemical processes in humans [121], while they have also been found to stimulate immunological responses [122]. AKG have also demonstrated the potential to defend the human organism from the side effects of radiotherapy [123]. In oncology, the therapeutic potential of AKG is prominent and significant. For example, oral administration of AKG to mice grafted with tumor of Lewis Lung Carcinoma (3LL) caused a reduction in the spread of mitosis and the presence of tumors with von Willebrand factor (vWF), the marker of tumor vascular endothelium, therefore signifying the AKG’s antiangiogenic effect [124]. Strong inhibition of tumor growth in three human prostate cancers (DU-145, PC-3 and PCa-2b) was also reported in a study using in vitro administration of shark liver oil containing 20% of 1-0-alkyl-2-3-diacyl-*sn*-glycerols (DAGE) [121]. Moreover, the administration of AKG exhibited also antiproliferative effects against colon cancer [125]. Finally, shark liver oils rich in AKG and with low amounts of n-3-PUFA, exhibited beneficial anti-inflammatory modulation of the immune response through modification of the inflammatory PAF and diacylglycerol production, thus providing promising anti-cancer effects [119].

Fish oils and fish/shark liver oils contain n-3 PUFA and other lipid bioactives, like PL, AKG, squalene, and some lipid vitamins, which have also been reported to be responsible for several biological activities such as inhibition of tumor growth and enhancement of both macrophage activation and specific immunity in rodents and humans. Shark liver oil is currently gaining interest in the field of nutrition due to the presence of both n-3 PUFAs and AKG. Shark liver oil is also rich in vitamin A and tocopherol (vitamin E). Both vitamin A and vitamin E have antioxidant properties. These antioxidants protect the body from free radicals, which may accumulate and cause oxidative stress, making them potentially harmful molecules. Nevertheless, apart from their antioxidant potency, lipid vitamins found in fish and shark oils also possess several other important bio-functionalities and cardio-protective properties.

### 2.4. Lipid Vitamins in Fish

#### 2.4.1. The Lipid Vitamin D

Vitamin D is a group of fat soluble secosteroids. Vitamin D exhibits a number of physiological activities, mostly relating to calcium and phosphorus homeostasis, and skeletal function and structure. There are two forms of vitamin D that are physiologically important. The two forms differ in their side-chains. They are known as ergocalciferol (vitamin D2), which naturally occurs in plants, and cholecalciferol (vitamin D3), which is found in animal organisms, including fish [126].

Vitamin D is a key regulator in the transcellular uptake of calcium and induces cytosolic calcium transport [127]. Moreover, vitamin D also regulates the expression of Na+-dependent Pi transporters, which is the main transport route for the uptake of phosphate in the intestine and then reabsorption in the kidney [128]. Malfunctioning of the vitamin D endocrine system or gastrointestinal disorders may result in bone diseases [129,130]. Vitamin D influences osteoblast activity and osteoclast formation due to the large accumulation of calcium phosphate in bone [131].

However, vitamin D has recently been accredited to playing vital roles in several other biological processes unrelated to calcium homeostasis. The fact that most of the human cells and tissues contain vitamin D receptors further indicates the many extra-skeletal bioactivities of this vitamin, particularly in the immune and cardiovascular systems [132]. Recent research has emerged, investigating the role of vitamin D in muscle function, autoimmune diseases, and the overall cardiovascular physiology [129,132,133,134].

Vitamin D also affects cell proliferation and differentiation, while vitamin D analogues that have a strong effect on regulating cell growth and differentiation have also shown great promise as novel treatment for diseases involving unregulated cell growth such as cancer [135]. Vitamin D has also been proposed for cancer prevention, since it has prevented new cases of breast and colorectal cancer [136]. Moreover, both vitamin D and its analogues, including paricalcitol, have been found to possess strong anti-inflammatory and anti-thrombotic properties against the inflammatory and thrombotic PAF-associated pathways, while their administration in renal patients under haemodialysis resulted in favorable reduction of inflammation with several health benefits, by reducing PAF levels to homeostatic ones, which subsequently reduced the levels of associated PAF inflammatory cytokines like IL-1, IL-6 and TNFa [137].

Subsequently, vitamin D deficiency, defined as less than 25 nmol\L blood concentration of serum 25-hydroxy vitamin D [138], has been associated with a number of diseases and dysfunctions such as rickets, muscle weakness, osteoporosis, various autoimmune diseases (e.g., type 1 diabetes, psoriasis), hypertension, CVD and many types of cancer [126]. For example, vitamin D deficiency has been associated with various cardiovascular risk factors and appears to be linked to a higher mortality and incidence of CVD, while several mechanisms have been proposed, such as renin-angiotensin-aldosterone system activation, abnormal nitric oxide regulation, oxidative stress, or altered inflammatory pathways [132]. However, it should be noticed that the levels proposed for a beneficial effect of vitamin D administration on immune function with anti-inflammatory benefits against persistent infections, such as the coronavirus disease (COVID-19) pandemic caused SARS-CoV-2, vary from study to study and have not been yet fully clarified [139,140].

Nevertheless, the multitude of diseases promoted by deficiency of vitamin D explains the importance of providing the human organism with a constant and sufficient supply of vitamin D, which should be treated as a high priority. For example, moderate to strong associations have also been identified between lower serum vitamin D concentrations and stroke and cardiovascular events, even after controlling for traditional demographic and lifestyle covariates, while the mechanisms of these associations have been widely examined both in animal and human studies [141]. Optimization of vitamin D levels in human subjects may improve insulin sensitivity and beta-cell function and lower levels of inflammatory markers too [141].

Apart from producing vitamin D from skin exposure to sunlight via ultraviolet B, humans can obtain the significant levels of this vitamin from the diet as well, and especially from oily fish and fish oils, including fish liver oil, suggesting fish as an essential dietary source of vitamin D for humans [1,132,142,143,144]. Generally, fish reserve large quantities of vitamin D in their fat tissues and liver. As fish cannot synthesize vitamin D, they must therefore obtain it from dietary sources in order to meet their daily requirements. Planktonic vitamin D naturally accumulates in the aquaculture food chain [145], and thus fish incorporate vitamins D2 and D3 through the diet [146]. Nevertheless, it is inconclusive that fatty fish contain more vitamin D3 and D2 than lean fish, as there is no correlation between the vitamin D content and the levels of fat in the muscle tissue of fish [126]. Thus, intake of fish and fish oils that are also rich sources of vitamin D, are thus highly regarded for their health benefits and the development and growth in humans.

For instance, fish liver, fish liver oil, oily fish and seagull eggs have been major sources of vitamin D for the coastal population of Norway [147], with salmon being widely proposed as a good example of a fish source rich in both n-3 PUFA and vitamin D [148,149], while variations in vitamin D content and differences between wild and farmed salmon [149] also suggest further research to ensure a sustainable production of salmon with adequate vitamin D. Analysis of vitamin D content in various fish species indicated that the disproportion between requirement and supply seems too vast to enable eradication of vitamin D deficiency by fish food-based solutions. Still, increasing fish consumption or changing consumption patterns could be beneficial and result in noticeable improvements in vitamin D status [126].

For example, appropriate modification of fish feed can beneficially improve the fish lipid content and the overall intake levels of fish lipid bioactives, including n-3 PUFA and vitamin D3, as it was observed after consumption of two portions/week of salmon raised on rapeseed oil, which increased the n-3 PUFA index and the vitamin D status, and decreased plasma triacylglycerols in the study group, with nutritional and cardio-protective health benefits [150]. Moreover, vitamin D3 and n-3 PUFA levels were significantly increased in salmon that had a basal diet supplemented with 10% of spray-dried microalga *Nannochloropsis gaditana* (NG) [148], suggesting that spray-dried microalga like NG represent novel, functional, natural ingredients of fish feed and are a sustainable source of n-3 PUFA that can raise the levels of healthy fats and vitamin D3 in farmed salmon, and subsequently in salmon-based oils. Valorization of fish processing by-products is a good example of an alternative sustainable source of fish lipid bioactives, including vitamin D. For instance, belly flap derived from processing Norwegian spring-spawning herring was found to contain twice the amount of vitamin D, EPA, and DHA, compared to herring fillets [151].

A classic example of the benefits of fish and fish lipid bioactives’ intake against vitamin D deficiency and associated health issues is in pregnant women, where a strong association between vitamin D deficiency and various adverse pregnancy outcomes has been observed, due to the pregnant woman being the only source of vitamin D for the fetus [152]. For example, vitamin D deficiency during pregnancy has been associated with some adverse neonatal outcomes as well as an increased risk of late pregnancy complications. The main sources of vitamin D for pregnant women are sunlight, fortified dairy products, oily fish, and dietary supplements. A potential positive effect of vitamin D supplementation during pregnancy against the decreased risk of these complications has been proposed [152]. In addition, fish and fish products, including fish oil, showed the highest contribution to vitamin D intake (35.8%) in Malaysian pregnant women, as a dietary measure to reduce vitamin D deficiency, which unfortunately is prevalent in this population despite perennial sunshine. Subsequently, a higher intake of vitamin D was associated with lower odds of vitamin D deficiency among these pregnant women [153].

Moreover, fish and seafood intake resulted in higher n-3 PUFA and vitamin status, in a Canadian population studied from 2004 until 2015, [154], while in a study of 440,581 UK Biobank participants, regular consumption of oily fish was associated with reduced odds of vitamin D deficiency across all ethnicities (white European, Asian, black African, Chinese, and mixed ancestry) of this UK population [155]. Furthermore, the natural fish diet of coastal Kerala and the latitude seems to be protective against vitamin D deficiency in children and the overall population of this territory in India, where a low prevalence of vitamin D deficiency has been observed [156]. In contrast, no fish consumption, among other parameters, such as less sun exposure time, were among the significant and independent determinants of vitamin D deficiency in Chinese centenarians, especially in women of this population, [157], which further suggests the need of vitamin D intake from fish and fish products rich in vitamin D like fish oils.

In addition to that, the most crucial factors influencing vitamin D status in Polish women with endocrine and osteoporotic disorders, were mainly regular fish consumption, spending holidays in sunny destinations, and regular intake of vitamin D preparations [158]. In another sample of young Polish women, aged 15–30 years, herring, sardine, and tuna products were amongst the food sources where highest vitamin D intake was observed, with a strong correlation between total vitamin D intake and its intake from these fish sources, while the correlation between total vitamin D intake and the number of servings was strongest for herring, sardine, and tuna products, in comparison to all the other food sources [159]. At the same time, while compared with other fish species, consuming herring was the strongest predictor of meeting the recommended vitamin D level of 10 µg, but also of 5 µg, which further suggests that herring is a relatively low-cost fish species with a high vitamin D content that can also beneficially influences the total vitamin D intake [159]. It seems beneficial to recommend young women to increase their herring intake and/or fish lipid bioactives from herring, in order to increase dietary vitamin D intake and to meet its recommendations.

Sufficient serum vitamin D levels achieved in response to a dietary intervention of a Mediterranean dietary pattern with two fatty fish meals/week for six months in asthmatic children, modified the beneficial response of pulmonary function and enhanced ventilatory function [160]. A 12-months sardine-enriched diet in elderly population, with prediabetes, increased sardines’ characteristic nutrients, such as the n-3 PUFA, EPA and DHA, vitamin D, fluorine, and taurine, in the group supplemented with sardines twice per week, and further suggested a greater protective effect against developing type-II diabetes and cardiovascular events in this population [161]. Association of low vitamin D intake with dyslipidaemia and vitamin D insufficiency/deficiency has been observed in Brazilian children, whereas the food groups that contributed the most to vitamin D intake were dairy products and fish, suggesting specific actions that promote and facilitate access to vitamin D food sources, such as dairy products and fish [162].

Despite all these observed benefits of fish intake against vitamin D deficiency and associated diseases, including CVD, evidence from the latest randomized controlled trials have indicated no benefits of vitamin D supplementation for CVD, and thus vitamin D supplements should not be recommended for CVD prevention [132]. A characteristic example of an important trial investigating the beneficial effects of the supplementation of marine n-3 PUFA (1 g/d) or vitamin D (2000 IU/d) in the primary prevention of CVD and cancer among in general populations is the randomized, placebo-controlled, 2 × 2 factorial VITAL trial (Vitamin D and Omega-3 Trial), where it was shown that vitamin D supplementation did not reduce major CVD events or other cardiovascular end points, while updated meta-analyses that include VITAL and other recent trials document coronary risk reduction from supplemental marine n-3 PUFA but no clear CVD risk reduction from supplemental vitamin D [163]. Whether such supplements containing fish n-3 PUFA or vitamin D can prevent late-life depression was also investigated in the VITAL-DEP trial (VITamin D and OmegA-3 TriaL-Depression Endpoint Prevention), an ancillary to the VITAL trial [164], where among adults aged 50 years or older without clinically relevant depressive symptoms at baseline, treatment with n-3 PUFA based supplements compared with placebo yielded mixed results, with a small but statistically significant increase in risk of depression or clinically relevant depressive symptoms but no difference in mood scores, over a median follow-up of 5.3 years, suggesting that no benefits can be obtained by the use of omega-3 supplements in adults to prevent depression [165].

Although several such trials on the effects of n-3 PUFA and/or vitamin D supplementation against cardiovascular endpoints are in progress, these are mainly using pharmacological doses. Thus, in view of the potential toxicity of pharmacological doses, there remains a need for long-term trials of physiological doses of vitamins D2 and D3 with CVD incidence as the primary outcome, such as those obtained by oily fish (i.e., diets containing at least one portion of oily fish per week supply about 7 μg/d) [166], while other forms of n-3 PUFA supplementation, such as those of fish PL may provide better outcomes.

Nevertheless, a recent meta-analysis investigating the influence of fish consumption in randomized controlled trials (RCTs) on serum vitamin D3 concentrations, has indicated that fish is indeed one of the major food sources of vitamin D that can significantly increase concentrations of 25(OH)D, although the recommended fish intakes cannot optimize vitamin D status [167], which further suggests that supplementation of vitamin D in fish oil concentrates may also be beneficial. Thus, to secure adequate vitamin D status while keeping the intake of dioxins and dl-polychlorinated biphenyls low, a healthy intervention should include both supplemental vitamin D and oily fish or fish oils, while oils from fish sources like dietary fish liver may need to be restricted despite their high nutrient content, due to unwanted compounds and possible antagonism to vitamins A and D [147,168]. Overall, due to the wide range of benefits associated with vitamin D consumption/administration, the presence and inclusion of vitamin D in fish and fish oils provides further promising outcomes and future perspectives for novel added value products based on extracts of fish lipids that also contain vitamin D, among other fish lipid bioactives.

#### 2.4.2. The Lipid Vitamin E

Vitamins E and A are lipophilic vitamins present in several fish oils, including shark liver oils, with mainly strong antioxidant properties. Extensive research has been conducted on the benefits of the presence of vitamin E in fish and its importance for fish neural and overall development [169,170,171]. The vitamin E group comprises eight lipophilic molecules, tocopherol (α-, β-, γ- and δ-), and tocotrienol (α-, β-, γ- and δ-), which exhibit antioxidant effects by scavenging free radicals and singlet oxygen [172,173,174]. Neither vitamin E isomers are known to be synthesized in fish and so must be obtained from the diet [173]. For farmed fish species, insufficient levels of tocopherols in the diet can lead to poor growth performance or to nutritional disease, while the tocopherol quantity needed as a feed supplement depends on various factors, such as the vitamer mixture, the lipid level and source, the method of diet preparation, and the feed storage conditions [174]. Thus, the level of the content of all these vitamin E isomers in seafood and aquatic products varies greatly, depending on the species [173,175], since Antarctic fish were found to contain five to six times higher amounts of vitamin E than temperate water fish species as a protection in the biochemical adaptation of fish in cold-waters [173], as well as whether they are wild or farmed fish and in the farming process [174,175]. For example, a 100 g serving of salmon may provide nearly 14% of the recommended dietary allowance of vitamin E [174], while it has also been proposed that the consumption of different species of seafood and aquatic products can bring in different effects on total activity and intake of vitamin E isomers [174].

The strong antioxidant properties of vitamin E are associated with the main effects of its presence in fish and fish oils, which is to beneficially protect against lipid peroxidation of the long chain unsaturated fatty acids, and especially being prone to oxidation of n-3 PUFA, EPA, and DHA, in fish and fish oils [105,176]. Incorporation of exogenous tocopherols in extracted fish oils at low concentrations maintain the quality of both muscle and the extracted oils during food storage [176]. However, when tocopherols are included in a fish diet (endogenous tocopherols), the antioxidant effect on the muscle lipids is more effective due to their incorporation into the membrane lipids, which can help extend the shelf life of seafood by reducing the lipid deterioration [176]. The vitamin E isomer, α-tocopherol, is also present endogenously in several fish PL extracts too, protecting the rich in PUFA fish PL from oxidation, while a synergistic effect of vitamin E with the tight intermolecular packing conformation at the *sn*-2 position of fish PL, also allows the formation of stable liposomes of fish PL, which are attractive ingredients for food or feed applications [177]. Moreover, vitamin E prevents the build-up of oxidized vitamin E molecules due to its mechanism of being recycled by other natural antioxidants [178].

Nevertheless, tocopherols are important not only from a nutritional point of view but also from a physiological one, since they are involved in many metabolic processes in the human organism. For example, epidemiological studies have suggested that the intake of vitamin E prevents oxidation of LDL [179] and reduces the risk of CVD [180]. Apart from its antioxidant potency, vitamin E has many other vital roles, from boosting the immune system to preventing blood clots, and thus subsequently it has also been found to possess strong anti-thrombotic and anti-inflammatory properties [181,182,183]. Moreover, the administration in children on home parenteral nutrition in a pediatric intestinal rehabilitation unit of a fish oil containing mixed lipid emulsions (SMOFlipid^®^) with a higher vitamin E content, which possess a theoretical risk of exceeding current recommendations for vitamin E dosing, may beneficially influence other fat soluble vitamin status in these children, since such an administration was correlated with higher vitamin E level, while a lower vitamin D level appeared in the group that was not administered with the SMOFlipid^®^, which further suggests the benefits of a fish oil rich in the lipid vitamin E on the vitamin D status [184].

Concerning the cardiovascular benefits of vitamin E and fish oil, this has mainly been researched in studies investigating the co-administration of both, with one enhancing the beneficial effects of the other, with vitamin E also providing protection against oxidative stress. For example, administration of vitamin E and/or fish oil in high cholesterol-fed rabbits was found to attenuate atherosclerosis, while vitamin E and fish oil potentiated the effect of each other [185]. Even though n-3 PUFA and fish oil administration have been unfavorably associated with an increase of LDL-cholesterol concentrations, along with a potential of increased oxidizability of LDL due to adverse lipid modification by such an administration, vitamin E co-supplementation has also been found to overcome these unfavorable events, and thus facilitates the anti-atherogenic lipid modifications and an overall cardiovascular protection of the n-3 PUFA, such as the increased HDL(2)-cholesterol concentrations, reduced triacylglycerol-rich lipoprotein concentrations, reduced postprandial lipemia, and reduced remnant concentrations [186].

Associations of fish oil and vitamin E co-supplementation with cardiovascular outcomes and mortality in people receiving hemodialysis have also been thoroughly reviewed, suggesting that more robust trials are needed to establish the health benefits of such an administration [187]. In addition, the co-presence of fish oils rich in n-3 PUFA and vitamin D has also been proposed to provide several health benefits in other disorders and inflammatory manifestations, such as rheumatoid arthritis and dysmenorrhea pain [188,189]. For example, daily co-administration of fish oil rich in n-3 PUFA and of vitamin E in female students resulted in a considerable beneficial effect against menstrual pain, which further suggested that the anti-inflammatory potential and benefits of the co-presence of these n-3 PUFA bioactives with vitamin E are helpful in reducing dysmenorrhea pain and may replace nonsteroidal anti-inflammatory drugs that pose high complications [189].

Consumption of fish oils in elderly people have a potentially beneficial effect on age-associated diseases, whereas recent studies have suggested that increased intake of fish oils and dietary PUFA may increase the requirement for vitamin E too, especially under conditions where oxidative stress is increased. More specifically, older subjects may be more susceptible to oxidative damage from oxygen radicals and other products of free radical reactions involved in aging and age-related degenerative diseases in conditions where the percentage of the potentially unstable highly unsaturated fish fatty acids increases in the membrane by substituting other membrane fatty acids, while the supplementation of vitamin E provided an antioxidant protection against these inflammatory complications in elderly people [190]. Moreover, vitamin E supplementation in elderly people did not only not interfere but rather enhanced and contributed to the beneficial inhibitory properties of fish oil supplementation against the production of pro-inflammatory cytokines [191]. Thus, more research is needed to fully evaluate the effects of the co-presence of vitamin E in fish oils and fish lipid extracts.

#### 2.4.3. The Lipid Vitamin A and Marine Carotenoids

Vitamin A is a general term encompassing various fat-soluble molecules, including retinol, retinyl palmitate, and beta-carotene, while the various metabolites of vitamin A are essential for vision, immune function, and cellular differentiation [192]. The fat-soluble vitamin A is also present in various fish species, while several carotenoids, including marine ones, show vitamin A activity. For example, considerable amounts of retinol (vitamin A1) and the carotenoids astaxanthin and canthaxanthin important for pigmentation of the muscle were present in raw salmon, salmon trout, and trout [193]. Vitamin A content was also a considerable proportion of the total lipids’ content of raw wild sea bass, while in farmed raw seabass there was only trace amounts of vitamin A [194]. Nevertheless, the composition of diets devoted to marine fish larvae, and especially the content of fish-feed in vitamin A and n-3 PUFA, has a particularly determining effect on the subsequent development of larvae and juvenile fish [195].

The preformed vitamin A (retinol and retinyl ester) is derived from animal food sources, including fish [192], while provitamin A (beta-carotenoid) is mainly derived from colorful fruits and vegetables, including several marine carotenoids that can also induce vitamin A activities. Both ingested forms of vitamin A are converted to retinal and retinoic acid after absorption to support several biological processes [192]. Vitamin A deficiency (VAD) is a highly prevalent health concern associated with substantial morbidity and mortality, mostly affecting young children in impoverished regions throughout the world, where insufficient intake of sources of vitamin A, including fish, can lead to deficiency and compromise of essential physiologic processes [196].

Several vitamin A isomers have been found in fish, with retinol (vitamin A1) being the most investigated one, while other isomers like the 3,4-didehydroretinol (vitamin A2) have also been found in freshwater fish and were proposed to also play a significant biological role, especially in reducing vitamin A deficiency [197]. The functions of these vitamins A from fish have been investigated mainly for food strategies addressing vitamin A deficiency in developing and undeveloped countries [198]. In poor, rural, households of such countries, the frequency of intake of indigenous small fish is high [198]. Thus, in areas where affordable indigenous fish species with a high vitamin A content are readily available and consumed, with less cost, these fish can be a promising dietary source for reducing vitamin A deficiency [197,199]. For example, even a small production of the vitamin A-rich fish mola in ponds in Bangladesh can meet the annual vitamin A recommendation of 2 million children [198].

Even though vitamin A is present in many fish tissues and cells, it seems that fish liver accumulates the majority of fish vitamin A content, suggesting that fish liver oils are rich sources of vitamin A [200], including shark liver oil, which is also rich in vitamin A. Nevertheless, caution is needed in the consumption of fish liver oil with a high content in vitamin A or of food supplements rich in vitamin A, since several cases of hypervitaminosis A toxification from excess vitamin A intake can take place [201]. Moreover, pregnant women or women of child-bearing age should be informed of the risk to pregnancy in the case of excessive fish liver and fish liver oil ingestion, due to possible teratogenicity associated with hypervitaminosis A poisoning [201,202]. On the other hand, valorization of fish processing by-products is also an alternative sustainable source of fish lipid bioactives, including vitamin A. For instance, belly flap derived from processing of Norwegian spring-spawning herring was found to contain considerably equal amounts of vitamin A, compared to herring fillets [151].

Among fish and marine carotenoids, astaxanthin, a red pigment from the carotenoid group, have shown strong antioxidant properties and several other bioactivities, which however does not include the properties of a provitamin A. Salmon and trout are rich in astaxanthin from the algae and the fish food chain, while astaxanthin is usually a feed ingredient for farmed fish and as a food coloring [203]. Astaxanthin most effectively protects cells, lipids, and lipoproteins of cell membranes against oxidative damage. The antioxidant activity of astaxanthin is 10 times more than zeaxanthin, lutein, canthaxanthin, β-carotene, and 100 times higher than α-tocopherol. Thus, astaxanthin was more effective than fish oil in modulating the immune system response and reducing the risk of vascular and inflammatory disease, including atherosclerotic cardiovascular disease and cancer [203,204].

Overall, the presence of several bio-functional nutrients in seafood (fish in particular), including fish lipid bioactives, such as n-3 PUFA, tocopherols and the lipid vitamins D and A and carotenoids like astaxanthin, seems to be associated with several beneficial effects of these ingredients on blood pressure, lipid profile and the inflammatory processes of chronic diseases, such as CVD, cancer, diabetes and mental disorder, chronic respiratory diseases are common diseases associated with advanced age [1,6,29].

Several epidemiological studies and clinical trials have shown that fish and its lipid bioactives reduce the risk and incidences of CVD and several other chronic disorders. However, several studies mention fish and fish oil benefits, which however have undergone specific thermal procedures such as cooking. Apart from fish that is served raw in preparations such as sushi and ceviche, fish is normally consumed cooked, while fish oils are usually produced after using a “cooking” process and thermal processing. The paradigm of the effects of various different types of cooking techniques on the main fish lipid bioactives, as presented in Table S2, provides the evidence of conformational and structural changes of fish nutrient content, lipid profile, and functionality, thereby affecting the health benefits of fish lipid bioactives, fish oils, and relative supplements. Therefore, evaluating the effects of different cooking methods and thermal processing such as steaming, frying, boiling, baking, and more mild emerging techniques like the sous-vide technique, on fish lipid content, bio-functionality, and activity of the most common commercial fish is of great importance.

## 3. The Effects of Thermal Processing—Cooking on the Bio-Functionality of Fish Lipid Content

Fish is a significant source of lipid bioactives, possessing many health benefits, including anti-inflammatory, anti-thrombotic, and cardioprotective ones [6,29,32]. However, due to consumers’ requirements and several safety issues such as bacteria contamination, fish is mainly consumed after thermal processing (i.e., cooked fish), while several extraction procedures for producing fish oils and relative supplements are based on thermal processing. An extensive review on the effects of several cooking methods and thermal conditions applied on fish on the lipid profile and bio-functionality of fish lipids is presented on Table S2. Research into how thermal processing, including cooking, can alter the lipid content and functionality in a variety of fish, is important, since it is a usual process needed for producing the fish lipid products such as fish oil and oily fish supplements, which among cooked fish are now consumed worldwide. It is also important to look into different species of fish, as fish from various ecosystems differ in their lipid content and how this can be affected by thermal processing. There has been some research into factors that can affect the lipid content in fish after thermal processing and cooking. These include cooking temperature and fish species, among others as well as surface contact and lipid content. However, more extensive research should be done as fish is now cooked in many ways.

Moreover, heat processes and temperatures cause different lipid molecules to behave differently. Saturated fatty acids (SFA) are heat stable until they reach a temperature of 150 °C. When this temperature is exceeded and there is oxygen present, oxidation takes place. Unsaturated fatty acids are more heat liable during thermal processing. Thus, considerable attention has been given to the effects of thermal processing like cooking on the lipid content of fish and especially on fish lipid bio-actives like the essential fatty acid ALA and the long chain n-3 PUFA, EPA, and DHA that are in significant quantities in fish, but also on the levels of the n-6/n-3 PUFA ratio and the fish polar lipids content and lipid vitamins found in fish [205].

During thermal processing, there are many reactions that take place both physically and chemically, which can alter the lipid content of the fish. Usually, fish lipid oxidation reactions occur, with free radicals being produced as fish is cooked. The application of heat, including cooking, conducted in a variety of ways, including conventional cooking and the use of a microwave, have been proposed to enhance the susceptibility of n-3 PUFA to oxidation. When talking about oxidation, it is vital to understand that various fatty acids can respond in different ways to heat treatment. Unsaturated fatty acids are normally more heat labile; but, as the degree of unsaturation increases, the stability decreases. Therefore, PUFA are the less stable fatty acid class. When combined with oxygen, the degradation of PUFA occurs more easily, and PUFA go through noticeable oxidative effects [206].

Heating also causes fat-soluble molecules to be leached and moisture to be lost from the fish tissues, while when cooking medium is used, then lipids from this medium may for instance be absorbed in the fish, causing either an increase in the amount of fatty acids present or a decrease in the fatty acid content, depending on which of the above mentioned processes is taking place [207].

The paradigm of the effects of cooking on fish lipids provide further evidence on some detrimental effects of thermal processing on fish lipid bioactives (Table S2). The cooking of fish, whilst making it safe to eat and aid digestibility, and the temperatures reached during the process, affect the lipid composition of the fish radically, leading to lipid oxidation. More specifically, different cooking methods on different species of fish prove to have different nutritional outcomes. Traditional cooking methods in high temperatures, such as frying, steaming, and pan-frying, are used all over the world, but emerging cooking methods in milder conditions have become more popular over the past decades, like the sous-vide cooking technique [208]. Pan frying is arguably the most popular method of cooking fish, as it is fast and convenient, while providing the desired effects in terms of flavor, color, and texture. However, there are some negative attributes to frying. Pan frying causes a change in the medium which it is fried in, but also the fish itself. Pan frying causes an elevated temperature, which can lead to degradation. Lipid oxidation can occur. Different oils in which the fish are fried in have different susceptibilities towards oxidative dilapidation because of the differences in their fatty acid unsaturation and their general lipid profile [205,206,207,209].

As a result, it has been hypothesized that traditional cooking methods degrade long-chain PUFA, EPA, and DHA due to the high temperatures created, which leads to the breaking of double bonds, resulting in oxidation of n-3 PUFA and thus to an unfavorable increase of the n-6/n-3 PUFA ratio. When n-6 is increased and n-3 is decreased, it can lead to increased prevalence and risk of CVD, obesity, and related chronic inflammatory diseases [19]. Apart from the n-6/n-3 PUFA ratio, changes were also observed in the PUFA/SFA ratio and in the fat-soluble vitamins A, D, and E, being present in fish, which are also prone to oxidation during heat treatments and cooking processes too [34].

There are many reasons for the possible changes. Some reactions take place while cooking, also including the absorption of the cooking fat, the leaching of fat-soluble molecules from the fish, together with oxidation reactions that generate free radicals in the hot cooking fat [210]. This may affect our health as a surplus of free radicles in our body may cause oxidative stress. This may result in the change of lipids, proteins, and DNA, which can then cause several diseases [211].

Cooking techniques that produce the lowest oxidation by-products are more beneficial for our health [205]. However, when high temperatures are sustained for the duration of these cooking processes, free radicals and reactive oxygen species (ROS) are more likely to form, resulting in the release of peroxides. When lipid peroxidation occurs, several unnecessary compounds are then created, such as alkanes, malanoaldehyde, and isoprostanes, which can lead to neurogenerative diseases [211]. Some of the oxidized lipids possess PAF-like structures and inflammatory activities. These PAF-like structures cause oxidative stress by mimicking the inflammatory and thrombotic activities of PAF, which causes platelet aggregation and blood vessel dilation, which in turn may lead to thrombosis, as well as several other thrombo-inflammatory manifestations implicated in endothelial dysfunction, atherosclerosis, CVD, and several tumors [28,29]. Therefore, the accumulation of these oxidized compounds can cause oxidative stress and trigger an inflammatory response [29].

Oxidation of n-3 PUFA results in a formation of unwanted by-products, such as hydroperoxides and aldehydes, which are highly toxic compounds. Aldehydes and various alcohol-derived compounds of the hydroperoxide primary metabolites are also released when further oxidation occurs. For example, lipid peroxidation induces the manufacture of reactive carbonyl compounds (RCCs) such as malondialdehyde (MDA), acrolein, or 4-hydroxy-alkenals, which include the 4-hydroxy-2-nonenal (4-HNE), a nine-carbon lipid aldehyde issued from the peroxidation of n-6 PUFA, and 4-hydroxy-2-hexenal (4-HHE), a six-carbon lipid aldehyde issued from the peroxidation of n-3 PUFAs. These unwanted by-products, produced by lipid oxidation, along with the peroxide value (PV), are the most common indexes for monitoring the effects of cooking on the lipid content of foods including oily fish like salmon [212].

After steaming saithe fillets, a rise in hydroperoxide levels was observed [213], indicating thermally catalyzed oxidation taking place during this thermal processing [214]. Atlantic salmon fried in olive or corn oil at 180 °C increased the peroxide value (PV) by three or two times, respectively, whilst rainbow trout fried in sunflower oil almost doubled the PV value [209]. High temperatures that are generated during various cooking procedures increase the free radicals and ROS formation and eventually lead to the release of various products such as peroxides [205].

When further oxidized, secondary products, namely aldehydes or alcohol-derived compounds of the hydroperoxide primary metabolites, are formed. F2-isoprostanes, which are prostaglandin-like derivatives of arachidonic acid, are examples of such metabolites formed during the cooking process. When all these compounds are formed it is generally regarded also as biomarkers for oxidative stress, which can then lead to a variety of diseases including endothelial disfunction, atherosclerosis, and CVD [194,205,215,216].

Such oxidized lipids (isoprostanes, PAF-like lipids, ROS, and aldehydes) host a major role in atherogenesis and can consequently play a role in both insulin resistance and vascular injury [29,217]. Ingestion of oxidized lipids in the form of fish oil dietary supplements can cause an increase in circulating oxidized lipid levels that are associated with increased cardiovascular risk in patients with coronary disease [218]. Oxidized lipids associated with low-density lipoprotein (LDL) contribute to endothelial dysfunction, atherosclerotic foam cell formation and inflammation [219], with small dense LDL (sdLDL) being considerably more atherogenic in comparison to larger LDL particles [220].

Nevertheless, different cooking techniques seem to similarly or differently affect the lipid content of different edible fish species [194,205,215]. For example, apart from the changes that may be observed during cooking on the overall PUFA content and the ALA, EPA, and DHA contents, the evaluation of the change of the n-6/n-3 PUFA ratio is also important during cooking, since, dependent on the cooking technique and the fish species, an increase of the n-6/n-3 PUFA ratio may occur from healthy anti-inflammatory levels (lower than 3/1) to westernized unhealthy ones (>6/1), which this may increase the risk for inflammation related chronic disorders, which include obesity, metabolic syndrome, CVD, and cancer [19].

Apart from the production of oxidized lipids and other unwanted by-products during lipid peroxidation when fish is under heat treatment, more harmful compounds can also be produced from oxidation of fish proteins too, such as the heterocyclic aromatic amines (HCA). There are many epidemiological studies linking this type of chemically produced molecules in fish treated at high temperatures, with several cancer forms [221]. Most HCAs appear to be mutagenic and, in 1993, the International Agency for Research on Cancer determined that almost all HCAs were carcinogenic [221,222].

On the other hand, paradigms of mild thermal processes like green extractions and sous-vide cooking have been proposed to have minor effects on the bio-functionality of the fish lipid content and lower production of unwanted and harmful by-products. For example, sous-vide cooking in terms of HCA has an advantage over other traditional cooking methods as it has relatively mild cooking temperatures. Sous-vide cooking has become well known due to its mild cooking conditions on fish and other products. It cooks food with a precise temperature in heat stable pouches while also keeping certain desirable flavors and other qualities. In their study, the aim of Wan et al. (2019) was to observe the effect sous-vide cooking has on the quality of largemouth bass (*Micropterus salmoides*) [223]. As a marker to understand the extent of oxidation, they used Thiobarbituric acid (TBA). This was due to TBA allowing the degree of oxidation that took place, shown by the amount of secondary oxidation products (MDA). When compared with the raw sample, the TBA value was increased. However, the TBA value in the sous-vide samples was lower (*p* < 0.05) than the boiled and steamed samples, which shows that due to a vacuum-like treatment, lipid oxidation was not as strong. The lesser the lipid oxidation, the more benefit it is for us to consume such thermal processed fish products, as there are less detrimental effects, and it therefore proves to be a better cooking method. This could be because the vacuum-like sealable pouch is able to isolate oxygen, which avoids the biochemical reactions that need oxygen [223].

As a plastic barrier is being used in this process, it limits the diffusion of oxygen to the food. This in turn prevents lipid oxidation, which is a benefit, as PUFAs will be preserved, and they provide major health benefits in human nutrition [224]. The oxidation of unsaturated fatty acid does not occur as frequently in sous-vide fish compared to other conventional methods of cooking at higher temperatures [225]. There was a higher content of unsaturated fatty acids present in the sous-vide samples compared to conventional cooking. The content of EPA, DHA, and DPA and the low values of the n-6/n-3 PUFA ratio of the PL of cooked salmon were retained during cooking sous-vide at low temperatures (50–65 °C), in which the antithrombotic potency of salmon PL was also retained, in contrast to salmon cooked sous-vide in higher temperatures, such as 80 °C [226]. Thus, the paradigm of sous-vide cooking further indicates that mild heat treatments do not affect substantially the n-3 PUFA content and some beneficial properties of fish lipids, while it can also reduce the creation of chemicals as heterocyclic amines and polycyclic aromatic hydrocarbons, which can be harmful to human health. There have also been studies to show that the vulnerable vitamins, such as the water-soluble vitamins B and C, are still present after sous-vide.

Therefore, by treating fish in low temperatures in this way, fish lipid bioactives, and especially the fish PL, are less susceptible to oxidation. As sous-vide uses low cooking temperatures it also prevents the break-up of compounds such as vitamins and antioxidants through solubilization and volatilization, while the anti-inflammatory and anti-thrombotic bioactivities of PL from salmon cooked sous-vide were sustained against inflammatory and thrombotic mediators such as PAF, as well as against well-established thrombotic factors like thrombin, ADP or collagen, both when low temperatures were applied (52 °C and 65 °C) or even when high temperatures for sous-vide were applied, such as those used for pasteurization (80 °C) [60]. Variations in the fatty acid content of PL in all sous-vide salmon preparations, especially specific PUFA, appear to be linked to the antithrombotic potency recorded. Changes in the amount of n-3 (DPA), for example, tend to be due to variations in the antithrombotic potency of PL from different sous-vide salmon preparations. Changes of the amounts of n-3 DPA present when prepared with different temperatures can affect the anti-collagen activities of salmon PL, as it is possible that PL rich in DPA can preferably inhibit the collagen pathway. The highest amount of n-3DPA found in the sample were samples prepared at 65 °C; however, it had decreased amounts present in both lower and higher temperature samples [60]. Nevertheless, the low values of the n-6/n-3 PUFA ratio in the PL of all sous-vide preparations, along with the strong inhibitory bioactivities of sous-vide cooked salmon PL against PAF, collagen, thrombin, and ADP, additionally propose a valuable role for such a mild cooking procedure for conserving the antithrombotic and cardio-protective properties of salmon without affecting its sensory characteristics.

There have been numerous experiments on different fish and different heat treatment processes. Some evidence is contradicting, so it is important to consider several factors when looking at results. In terms of microwaving and grilling, this has increased greatly in recent years and is not as harsh as it once was, thus outdating some of the previous studies conducted decades ago [227]. Moreover, some studies reported large changes to the PUFA and SFA as well as n-6/n-3 PUFA ratios after thermal processing and cooking, which contradicts other studies that have failed to see any major effects of the different cooking methods on fish fatty acid composition [228].

In some studies where no changes on the fish n-3 PUFA content were observed during heat treatments and cooking, it was proposed to be caused by a high level of natural fish antioxidants, which were developed during evolution as adaptation to their ecological niche. An example of such an antioxidant in Salmonidae is the carotenoid astaxanthin, which goes to fish from microalgae through the food chain. This is an important bio-functional lipid molecule with several proposed health benefits, such as the beneficial effects against neurodegenerative diseases [229]. In addition, it has also been reported that squalene, which is an antioxidant found in extracts of polar lipids, also protects against the oxidation of fish fatty acids [230].

Moreover, fish PL seem to be less affected by thermal processing and oxidation. For example, intense heat treatment did not affect the cardio-protective bioactivities of sea bream PL against the platelet aggregation induced by the highly bioactive inflammatory mediator platelet activating factor (PAF) [231]. In addition, the anti-inflammatory and antithrombotic bio-functionality of PL baring PUFA was not affected by mild heat treatment during the sous-vide technique in pouches [60]. Evaluation of the effects of grilling and brining on the sensory characteristics, the fillet fatty acid composition and the cardio-protective activity of PL from sardine (*Sardina pilchardus*) was also conducted [117]. It was found that the PL from the sardines undergone heat treatment were 8 times less biologically active against the inflammatory mediator PAF, than the raw sample [117]. Moreover, cod PL retained their strong anti-inflammatory and anti-atherogenic properties against the PAF-pathways during frying in sunflower oil, while in some cases this anti-inflammatory potency was even increased, maybe due to the absorbed lipid bioactives from sunflower oil to the fried cod [118].

There are very few studies that have investigated the fat-soluble vitamins after heat treatments. For example, changes were observed in the fat-soluble vitamins A, D, and E, being present in fish, which are also prone to oxidation during heat treatments and cooking processes [34]. A “twin fillet” approach was also used to determine the effect of fish heat treatments on the retention of compounds beneficial for humans to consume, such as fatty acids and fat-soluble vitamins (A, D3, and E) [232]. Vitamin A is proposed to be stable under an inert atmosphere when heated. However, the presence of oxygen could cause vitamin A quantities to degrade, while different cooking methods could cause a difference in the vitamin A quantities [232]. Vitamin A retinol maintained a retention considered quite high during heat treatment. It had an average of 79.03% for the cod that was fried. This leads us to believe that vitamin A is relatively stable in fish undergoing heat treatments. The procedure of cod frying was the only one for which true retention was substantially higher than apparent retention. The reason behind this is most likely due to an increase in the dry matter content of the cod, which could be due to water being lost and oil being absorbed. Overall, vitamin A is increased during grilling and frying, whereas reduced during baking and microwave heating [232].

The effects of heating on Vitamin D are also not thoroughly studied, as there are not sufficient studies to explicitly specify the effect of different cooking methods on the decomposition degree of vitamin D and its stability under heating [232]. Presumably, the level of vitamin D loss depends on the type of foodstuff and the heat process used [232]. For vitamin D3, steaming was the heat process which retained it the most. Its retention for cod was 102.44%. The frying process of cod resulted in the retention of vitamin D3 being 69.44%. This would mean that for fat soluble vitamins, steaming would be preferable [232]. No significant retention of vitamin D3 was also observed in rainbow trout after several thermal processing and cooking techniques, except for panfrying [233].

In terms of vitamin E, it is expected that it is not stable and would be lost during heat treatments. This is thought to be because of the structure of vitamin E being highly unsaturated, which leads to antioxidant activity, meaning that vitamin E is very liable to oxidation and thus protecting other molecules from being oxidized [232]. Although vitamin E is thought to be unstable, it proved differently in this study. It remained relatively stable during steaming and baking processes, similar to vitamins A and E. When it was undergoing baking, it reached 93.28%. When frying, there was an increase in the content of vitamin E. This was due to a high quantity of vitamin E present in rapeseed oil, which was the frying medium used [232]. The preservation of vitamin E in fish during cooking and extraction processes is highly important.

On that account, knowledge of the lipid content and functionality in several sustainable fish species is needed to fully understand and re-evaluate the utilization of fish lipids for fish oil and novel supplements and nutraceuticals. Thus, the paradigms of the effects of traditional and emerging thermal processing and cooking methods on the fish lipid content and lipid bioactives of the main oily fish species, such as salmon, mackerel, seabass, seabream, sardines, and herrings, but also to a white fish species like cod, which are mostly consumed worldwide and used for the production of fish oils and PUFA based supplements, are thoroughly presented in Table S2. Emphasis is given to the importance of changes occurring during different cooking techniques and thermal conditions applied on the fatty acid composition and alterations on the SFA, MUFA, PUFA content, and the n-6/n-3 PUFA ratio, as well as on the anti-inflammatory fish polar lipid bioactives and on the important lipid vitamins and carotenoids occurring in fish, fish oils, and fish-derived food supplements and nutraceuticals.

Based on the outcome that mild heat treatments, such as the ones applied in mild cooking techniques in water baths (sous-vide), which showed the mildest effects on the lipid content and functionality of fish lipid bioactives, followed by boiling and steaming as more suitable heat treatment nutrition-wise, shows that the milder the method for heat treatment in fish the lower the loss and oxidation of the fish lipid bioactives, which further emphasizes the need for greener methods of extractions of fish lipids, with none or the mildest cooking methods applied, for the preservation of the anti-inflammatory and overall health benefits of the extracted fish lipid bioactives. In addition, the presence of natural antioxidants in thus extracted fish lipid bioactives, such as squalene and marine carotenoids like astaxanthin, further protects against fish lipid oxidation and stability of the fish lipids [230], and further provides health benefits associated with their antioxidant properties [229].

## 4. Current and Future Perspectives on Green Extraction Methodologies for the Recovery of High-Quality Fish Lipid Bioactives

Traditional fish oil production at an industrial level also incorporates the “cooking” step, in which heat treatment is usually employed to extract and separate the lipids from fish meat and water. However, the paradigms of the chemical changes and the oxidation/peroxidation of the labile fish lipid bioactives occurring during such cooking and thermal processing, with the production of unfavorable compounds in fish and thus in the final fish oil extract, compromises fish oil quality and further indicates the need for incorporating new technologies for the production of fish oil, with less thermal processing. Furthermore, conventional extractions of fish lipids by using organic solvents provides satisfactory yields obtained, with, however, lengthy processes, high solvent consumption, the use of mostly toxic solvents, or with conditions that can degrade the quality of the extracted valuable compounds [36].

It is now necessary to explore viable and more sustainable processes, with less to zero thermal processing. In the case of using organic solvents, these should be food grade ones according to relevant legislations (i.e., EU legislations). This has led the research to focus on the development of alternative processes according to the concept of “green” extraction methods, ensuring the purity and stability of fish oil and products containing fish lipid bioactives (i.e., supplements and nutraceuticals), as well as minimizing the usage of non-food grade solvents, energy consumption, and environmental impact [234].

Several green extraction processes have been developed to address these issues, with supercritical fluid extraction (SFE), ultrasound assisted extraction (UAE), pulsed electric fields (PEF), and microwave assisted extraction (MAE) being the most promising ones. These green methods and their benefits and drawbacks have recently been thoroughly reviewed by Pateiro et al. (2021) and Khawli et al. (2019) [36,234], while SFE has been demonstrated by many as being the most widely environmentally friendly technology, which can also successfully be utilized for the recovery of lipid bioactives from marine side streams too. By applying this alternative green extraction method, a high content of nutritionally relevant fish lipid bioactive ingredients can be achieved, with reduced environmental impact [235]. Nevertheless, within the SFE method, heat treatment with mildly increased temperatures of 50–70 °C for 2–3 h are also applied, and thus further research is needed to evaluate the effects of this mild thermal processing on the quality of fish lipid bioactives.

Moreover, even though UAE has provided enriched oils with high added value compounds, there are still studies indicating that this technology could enhance lipid oxidation in the presence of oxygen, and thus some authors have applied UAE processes under a nitrogen atmosphere or with the addition of antioxidants to reduce the risk of lipid oxidation and loss of quality in the obtained oil [236,237], which however increases the cost for this application. PEF can also enhance the extraction of fish lipid bioactives while maintaining the oxidative stability of the obtained fish oils due to inactivating oxidative enzymes present in the fish [235,238,239], and can also be applied as a pretreatment or in combination with other techniques, including UAE, to increase the yield and reduce the risk of oxidation [240]. MAE is another method used either as pretreatment or in combination with other traditional techniques for increasing the yield of extraction and reducing the risk of oxidation of the extracted fish lipid bioactives and fish oils [241].

Moreover, recently it was also reported that fish oil rich in long-chain polyunsaturated fatty acids, vitamin D3, and carotenoid pigments have been sustainably extracted from anchovy fillet leftovers using a solid-liquid extraction based on citrus-derived d-limonene as a “green” bio solvent, as an alternative green method of fish oil extractions and microencapsulation [242,243,244]. The presence of this “green” terpene, which is also an antibacterial and antioxidant, protects PUFAs in the fish oil from otherwise quick catalytic oxidation due to free radicals formed in the presence of oxygen, both during the fish oil extraction at room temperature and during solvent recovery by evaporation under reduced pressure at 90 °C [244]. In addition, spray drying of a fish oil submicroemulsion stabilized by food-grade hydrophilic silica particles protects the n-3 PUFA from oxidation regardless of the harsh fabrication conditions (inlet temperature, 140 °C, and high pressure) employed to spray dry the functionalized silica particles [245], while encapsulating the newly extracted fish oil obtained from anchovy filleting industrial waste in periodic mesoporous silica particles, resulted in a fish oil rich in not only EPA and DHA, but also in vitamin D3 and natural carotenoids [243].

In addition, another example of “green” extractions for obtaining fish lipid bioactives, is the food-grade extracted salmon PL based on food-grade “green” solvents of the consolidated Directive 2009/32/EC of EU for food-grade extractions, for which the yield of extraction was the same as the one obtained using conventional organic solvents, while the anti-inflammatory and anti-thrombotic bioactivities of the salmon PL were retained using such food-grade extraction techniques [31]. Double blind, cross over, and placebo controlled human trials investigating the in vivo anti-inflammatory and anti-thrombotic cardio-protective benefits of the administration of such food grade salmon lipid extracts rich in fish PL bioactives have already been registered on the ClinicalTrials.gov (i.e., ClinicalTrials.gov Identifiers: NCT03603769 and NCT03866265) and their health outcomes will be released soon.

These alternative green methods are still under development and require additional research for finding ways to reduce their high costs and the costs of the initial investment that limit their use in the fish oil industry, while simultaneously increasing the efficacy of these promising environmental-friendly alternative techniques both on the yield and on the quality of the obtained fish oils and supplements.

## 5. Conclusions

Inflammation is widely regarded as the pathological link between chronic diseases such as metabolic-syndrome, obesity, diabetes, cancer, renal disorders, neurodegenerative disorders, inflammatory manifestations during persistent infections, and especially atherosclerosis and CVD. There is a common crosstalk between various inflammatory mediators like lipid mediators (eicosanoids, PAF, etc.), cytokines, growth factors, and adhesion molecules and their associated signaling pathways, which induce several underlying inflammatory processes and manifestations in inflammation-related disorders. Healthy dietary interventions, including those that incorporate the intake of fish and fish lipid bioactives (i.e., fish oil or supplements containing fish lipid bioactives) have shown a vast number of anti-inflammatory health benefits with no noticeable side effects.

Fish is a staple component of the healthy diets, such as the Mediterranean diet, and its richness in several bio-actives, including high biological value proteins, peptides, and bioactive lipid molecules, such as n-3 PUFA, lipid vitamins, and bio-functional fish polar lipids (PL) have shown to mediate the cardioprotective effects of fish via the lowering of inflammatory markers, triglycerides, oxidative stress, blood pressure, and improvement of vascular function. Main attention has been given to n-3 PUFA, such as the essential ALA and the long chain EPA and DHA, and products rich in n-3 PUFA (fish oil and supplements), while the benefits of fish lipids is likely to be through the interplay of a number of lipid bioactive nutrients found in fish and not just due to their high n-3 PUFA content. Recent evidence has indicated that the variety of lipid nutrients contained within the fish, such as the lipid vitamins and the bio-functional fish PL like marine phospholipids and glycolipids, and especially those baring n-3 PUFA within their structures, have also shown to exert cardioprotective properties, helping reduce CVD risk, while they also ameliorate other inflammation-related disorders like tumor metastatic procedures and neurodegenerative disorders. Overall, knowledge of the specific lipid composition of different fish species is also important. Within this study, emphasis was given to the most representative examples of commercially preferred oily fish, moderate fatty fish, and lean fish, like salmon, sardines, herring, sea bass, sea bream, and cod. The specific fatty acid composition (provided in Supplementary Table S1 [9,30,34,71,117,206,230,246,247,248,249,250,251,252,253,254,255,256]), the overall lipid content, and functionality for these fish species is thoroughly reviewed in this study.

As fish contain many lipid bioactives affected by heat treatment, which is one of the main steps for the production of fish oils and supplements, it is important to evaluate the paradigms of the effects of several cooking procedures and cooking mediums on fish lipid content and bio-functionalities. Several studies have highlighted that the application of heat, including cooking, conducted in a variety of ways, have been reported to increase the susceptibility of lipids to oxidation, and thus to reduce the levels and functionality of fish lipids and also to increase the n-6/n-3 PUFA ratio to unfavorable levels that can increase the inflammatory risk for chronic disorders (>6/1, with a usual value of 15–20/1). Heat treatment also tends to upsurge the formation of unwanted by-products such as, free radicals, ROS, peroxides, aldehydes, various alcohol-derived compounds of the hydroperoxide primary metabolites, and reactive carbonyl compound, which are also implicated in oxidative stress and inflammation-related manifestations and chronic disorders (these effects are summarized in Supplementary Table S2 [34,60,117,118,194,205,206,207,209,210,223,230,231,232,246,247,249,251,253,255,256,257,258,259,260,261,262,263,264,265,266,267,268,269,270]).

Thus, milder cooking techniques, and especially modern “green” technologies, are needed to provide yields of high-quality fish lipid bioactives for developing added value novel fish oils and supplements that are not only based on n-3 PUFA, rather than in the presence of other fish lipid bioactives too. Finally, since higher demands of fish and its highly-valued lipid bioactives (n-3 PUFA, bio-functional PL, and lipid vitamins) can lead to non-sustainable over-fishing, future research is also required for using sustainable fish sources, such as fish, aquaculture, and fisheries by-products, the valorization of which for bio-functional lipids can also lead to novel fish products with higher nutritional value and health benefits.

## Figures and Tables

**Table 1:** Proposed anti-inflammatory, anti-thrombotic, and cardio-protective health benefits of fish and fish lipid bioactives.

Reference	Study Design	Fish/Fish Oil/Fish Lipid Bioactives (Dose/Amount per Day in Cases of In Vivo Trials)	Health Effects Studied	Cell-Models (In Vitro)—Participants (In Vivo) (Duration)	Main Effects on Health	Other Health Observations—Benefits
[41]	Prospective study	n-3 PUFA	Risk of CVD	57,972 participants (12.7 years)	Reduced risk of mortality	Lowered blood pressure and inflammatory markers
[42]	Randomized crossover feeding trial	Salmon (113 g, twice/wk)	Incident of CHD	25 participants (4 weeks)	Lower cholesterol and triglyceride conc.	Increased HDL-cholesterol
[43]	Randomised controlled trial	Fish	Secondary prevention of MI	2033 participants (2 years)	29% reduction all-cause mortality	3–4% lower serum cholesterol
[44]	Randomised, placebo-controlled trial	Fish oil	Prevention of MI	122, 120, and 118 patients (1 year)	Decrease in total cardiac events	Reduced left ventricular enlargement and angina pectoris
[45]	Randomised controlled trial	n-3 PUFA	Prevention of MI	11,324 participants (3.5 years)	Lowered risk of primary endpoint	Reduced cholesterol and triglyceride
[46]	Randomized, double- blind, placebo-controlled clinically controlled trial	Fish oil concentrate (6 g/d for 3 months and 3 g/d for 21 months)	Effect on CHD	223 patients (2 years)	Lowering in CHD events	Loss in minimal luminal diameter
[47]	Meta-analysis	Dietary and non-dietary intake of n-3 PUFA	Effect on CHD	7951 participants in the intervention, 7855 participants controlled (1966–1999)	Reduction in overall mortality	Reduction in MI and sudden death
[48]	Cross-sectional study	n-3 PUFA	Effect on inflammatory biomarkers	1024 patients (2 years)	Inverse association of n-3 intake and levels of inflammatory biomarkers	
[49]	Epidemiological study	n-3 PUFA	Effect on inflammatory markers	1123 patients	Intake associated with lower levels of pro-inflammatory markers	Intake associated with high levels of anti-inflammatory markers
[50]	Cross-sectional study	n-3 PUFA and fish	Effect on inflammation and its related markers	5677 men and women	Lowered levels of inflammation and endothelial activation	Intake inversely associated with IL-6 levels
[51]	Cross-sectional study	n-3 PUFA and fish	Effect on low-grade inflammation, atheroclerosis and CVD	2000 participants	Inverse association with inflammatory marker levels	Triglycerides decreased across n-3 tertiles
[52]	Cross-sectional study	Fish	Inflammatory markers	3042 men and women	Associated with lower inflammatory marker levels	Significant results attained even in lower quantities on fish consumed
[53]	Meta-analysis	Fish oil	Inflammatory markers	7 trials included	Decreased levels of TNF-a and IL-6	C-reactive protein not significantly affected
[54]	Quantitative analysis	Fish	CHD mortality	8 studies	Reduced risk of CHD	3.9% reduction associated with each additional serving per week
[55]	Meta-analysis	Fish	CHD mortality	11 eligible and 13 cohort studies (11.5 years average follow up)	Inverse association with CHD mortality	Benefits achieved by consuming fish just once per week
[56]	Randomised controlled trial	Mediterranean diet supplemented with fatty fish	Inflammation in paediatric asthma	64 children (effects noticed after 6 months)	Reduced airway inflammation in childhood asthma	
[57]	Randomised controlled trial	Mediterranean diet supplemented with fish oil	Mental health	95 participants (6 months)	Improved mental health in people with depression	At 3 months significant inverse correlation between Med-scores and depression
[58]	Cross-sectional analysis	Fish	Rheumatoid arthritis	176 participants	Lowered disease activity and risk for CVD in RA patients	
[59]	Meta-analysis	EPA+DHA	Blood pressure	7 RCTs (2012–2014)	Reduced systolic blood pressue	>2 g reduces diastolic blood pressure
[60]	In vitro study	Polar lipids from salmon under thermal treatment (cooking) versus raw untreated salmon	Anti-inflammatory and anti-thrombotic properties	Human platelets	Εffects of thermal treatment on the anti-inflammatory and antithrombotic potency of salmon polar lipids	Salmon PL rich in n-3 PUFA retain their ability to inhibit human platelet aggregation induced by the inflammatory and thrombotic mediators PAF and thrombin, but also by well-established platelet agonists such as ADP and collagen, after heat treatment
[9]	In vitro study	Fish by-products	Anti-inflammatory and anti-thrombotic properties	Human platelets	PL from fish by-products inhibited human platelet aggregation induced by the inflammatory and thrombotic mediators PAF and thrombin, but also by well-established platelet agonists such as ADP and collagen	PL bioactives from fish by-products are putative candidates for the sustainable development of novel supplements and nutraceuticals with cardio-protective properties
[30,31]	In vitro study	Salmon PL	Anti-inflammatory and anti-thrombotic cardio-protective properties	Human platelets	Food grade extracted salmon PL bioactives inhibited human platelet aggregation induced by the inflammatory and thrombotic mediators PAF and thrombin, at the same levels as the conventional extracted salmon PL	Food grade extracted PL bioactives rich in n-3 PUFA from fish sources are putative candidates for developing novel supplements and nutraceuticals with cardio-protective properties, according to EFSA and EU legislations, in contrast to conventional extracted salmon PL
[26,61]	Ex vivo trial in hypercholesterolaemic rabbits	Fish polar lipids	Formation of Atherosclerotic plaques Serum Lipid profile Inflammatory levels and metabolism of PAF	12 rabbits (fish polar lipids were included in the diet of 66 rabbits versus another 6 that were not administered fish polar lipids (control) (45 days)	Evaluation of anti-atherogenic properties of fish PL: rabbits fed with hypercholesterolemic diet with fish PL developed atherosclerotic lessions of lower degree than the control ones, which were fed a hypercholesterolemic diet without the presence of fish PL. The inclusion of fish PL in the diet of these rabbits increased HDL levels as well	Fish PL modulated the metabolism of the inflammatory and thrombotic mediator, PAF, towards a reduction of PAF-levels to homeostatic lower levels in rabbits fed with hypercholesterolemic diet with fish PL, which reduced inflammation and thus reduced atherosclerosis progression
[62]	In vitro study	Sardine lipid bioactives and cod liver oil	Anti-platelet properties		Evaluation of the anti-platelet properties of an oily fish (sardines) and of a fish oil (cod liver oil) lipid bioactives as putative candidates for anti-atherogenic agents	Inhibition of rabbit platelet aggregation induced by the inflammatory and thrombotic mediator PAF
[63]	In vitro study	Fish lipids	Anti-platelet properties	Rabbit platelets	Evaluation of anti-platelet properties of fish lipid bioactives as putative candidates for anti-atherogenic agents	Inhibition of rabbit platelet aggregation induced by the inflammatory and thrombotic mediator PAF
[27]	In vitro study	Fish polar lipids	Inflammatory levels and metabolism of PAF	Human mesangial cells	Reduction of inflammatory activation of mesangial cells and thus reduction of risk for glomerulosclerosis and other kidney disorders	Effect on PAF metabolism towards reduction of PAF-levels to homeostatic ones, which reduced inflammation
[21]	Systematic review, Meta-analysis	Supplementation of n-3 PUFA	Risk of major cardiovascular disease events	20 studies—randomized trials that enrolled 68,680 patients throughout 2012	Lack of evidence to suggest the beneficial effect of n-3 PUFA supplementation in respect of cardiovascular events and other measurable changes in health	n-3 PUFA supplementation was not associated with a lower risk of all-cause mortality, cardiac death, sudden death, myocardial infarction, or stroke based on relative and absolute measures of association
[22]	Meta-analysis	Supplementation of n-3 PUFA	Risk of major cardiovascular disease events and complications in peripheral arterial disease (PAD)	Randomized trials throughout 2013 that enrolled 396 individuals and lasted more than 12 weeks in adults with PAD	Insufficient evidence exists to suggest a beneficial effect of n-3 PUFA supplementation in adults with PAD with regard to cardiovascular events and other serious clinical outcomes	There was no evidence of a protective association of n-3 PUFA supplementation against major adverse cardiac events or other serious clinical outcomes. Any adverse events and compliance were poorly reported
[23]	Systematic review	Supplementation of n-3 PUFA	Prevention of cardiovascular disease	2 meta-analysis studies on RCTs and 8 placebo-controlled RCTs, with more than 1000 patients and follow-up of more than a year, between 1999 and 2015, were included	There is currently a lack of evidence to support the routine use of omega-3 PUFAs in both the primary and secondary prevention of CVD. Safety of omega-3 PUFA supplementation should be considered and it was proposed that Pharmacists are ideally situated to engage patients in the discussion of the lack of benefit and possible risk of omega-3 PUFA	No reduction in CV events with n-3 PUFAs, in addition to standard, evidence-based therapy in patients after myocardial infarction. While data from RCTs have not demonstrated serious safety concerns, omega-3 PUFAs can increase the risk of bleeding and may interact with other medications that affect hemostasis, such as antiplatelet agents and warfarin
[24]	Meta-analysis	Supplementation of n-3 PUFA	Secondary prevention of cardiovascular disease	14 randomized, double-blind, placebo-controlled trials (involving 20,485 patients with a history of CVD) since April 2011	Insufficient evidence of a secondary preventive effect of n-3 PUFA supplements against overall cardiovascular events among patients with a history of cardiovascular disease	No reduction of the risk of overall cardiovascular events, all-cause mortality, sudden cardiac death, myocardial infarction, congestive heart failure, or transient ischemic attack and stroke
[25]	Systematic review, Meta-analysis	Consumption of fish and long chain n-3 PUFA	Risk of cerebrovascular disease	26 prospective cohort studies and 12 randomised controlled trials with aggregate data on 794,000 non-overlapping people and 34,817 cerebrovascular outcomes, were included	Μoderate, inverse associations of fish consumption and long chain omega 3 fatty acids with cerebrovascular risk. The beneficial effect of fish intake on cerebrovascular risk is likely to be mediated through the interplay of a wide range of nutrients abundant in fish	Long chain n-3 PUFA measured as circulating biomarkers in observational studies or supplements in primary and secondary prevention trials were not associated with cerebrovascular disease
[20]	Randomized, placebo-controlled trial, VITAL (Vitamin D and Omega-3 Trial)	A two-by-two factorial design, of vitamin D3 (at a dose of 2000 IU per day) and fish n-3 PUFA (at a dose of 1 g per day)	Primary prevention of cardiovascular disease and cancer	A total of 25,871 participants of men 50 years of age or older and women 55 years of age or older in the United States	Supplementation with n-3 PUFA did not result in a lower incidence of major cardiovascular events or cancer than placebo	
[35]	A population-based cohort study (the Singapore Chinese Health Study)	Dietary n−3 PUFA	Association with cardiovascular death	63,257 Chinese adults aged 45–74 years from 1993 to 1998	Higher intakes of marine (EPA/DHA) and plant (ALA) omega-3 fatty acids are both associated with reduced risk of cardiovascular mortality in a Chinese population. The associations were similar for deaths from CHD and stroke and persisted in participants who were free of CVD at the baseline	High dietary intake of both marine and non-marine-based omega-3 fatty acids is associated with reduced risk of cardiovascular death in the Chinese population, particularly for deaths from coronary heart disease and in individuals without cardiovascular disease at baseline The beneficial effects of fish consumption of CVD risk and other chronic disease is due to the interplay of an array of different lipid nutrients instead of just n-3 PUFA in their neutral form
[64]	A 2-by-2 factorial randomised control trial (Alpha Omega Trial), and no significant effect was found for either EPA/DHA or ALA	2 g ALA or 400 mg EPA/DHA as the interventions	Cardiovascular events after myocardial infarction	4837 post-myocardial infarction patients	No significant beneficial effect was found for all n-3 PUFA assessed (either EPA/DHA or ALA)	
[65]	Randomised control trial for Prevention of Post-operative Atrial Fibrillation (OPERA)	Peri-operative oral n-3 PUFA supplementation (8–10g of n-3 PUFA or placebo divided over 2–5 days followed by 2 g per day until discharged from hospital or post-operative day 10)	Reduction of the occurrence of post-operative atrial fibrillation	1516 patients receiving cardiac surgery	n-3 PUFA administration did not reduce the risk of post-operative atrial fibrillation, in comparison to the placebo	
[66]	Randomised control trial, ORIGIN (Outcome Reduction with an Initial Glargine Intervention)	Long term supplementation of n-3 PUFA (1g capsules per day, containing at least 900 mg of ethyl esters of n-3 PUFA)	Reduction of the rate of cardiovascular events in patients with either Type II diabetes, impaired fasting glucose or impaired glucose intolerance	12,537 participants during an average follow-up of 6.2 years	Incidences of death from cardiovascular causes did not decrease significantly amongst patients that received n-3 PUFA, in comparison to the control group that received the placebo	During >6 years of treatment followed by >2.5 years of observation, omega-3 fatty acid supplementation had no effect on health outcomes and salutary effects on metabolic control

Abbreviations: CVD = cardiovascular diseases; CHD = coronary heart disease; MI = myocardial infraction; RA = reumatoid arthritis; HDL = high density lipoprotein; TNF = tumor necrosis factor; IL-6 = interleukin-6; n-3 PUFA = omega-3 polyunsaturated fatty acids; EPA = eicosapentaenoic acid; DHA = docosahexaenoic acid; PL = polar lipids; PAF = platelet activating factor.However, in these products the bioactive n-3 PUFA are mainly bound in neutral esterified forms (i.e., lipid esters or TAG), with low bio-availability of their n-3 PUFA content, which are mainly driven towards the adipose tissue after ingestion, due to their more neutral and less polar nature. Thus, very high amounts of these neutral forms of n-3 PUFA bound in esters and TAG are needed in order to provide any anti-inflammatory and cardiovascular benefit. For example, according to the European Food Safety Authority (EFSA), 2–4 g/day of these neutral forms of n-3 PUFA are needed for any of these benefits, which is approximately 10 times higher than those achieved by moderate consumption of fish in healthy diets (0.25 g/day) [67]. The polarity of the final form of the bioactive n-3 PUFA plays a crucial role on their bioavailability and subsequently for the benefits observed from fish consumption rather than fish oils and supplements rich in esters of n-3 PUFA. The amphiphilic properties of PL allow for example this polar form of n-3 PUFA to pass barriers in our body that are difficult to be surpassed, such as the blood brain barrier, which the neutral forms of n-3 PUFA bound to TAG cannot easily pass [68]. Furthermore, the polar forms of n-3 PUFA in PL have more potent antithrombotic, anti-inflammatory, and cardio-protective properties than the neutral n-3 PUFA forms in TAG, and in much lower quantities [6,29,32].

## Supplementary Materials

The following supporting information can be downloaded at: https://www.mdpi.com/article/10.3390/md20030187/s1, Table S1: Characteristic examples on the fatty acid composition of important fish species (expressed in % of total fatty acids in TL or PL content of raw samples); Table S2: The effects of different thermal processing—cooking techniques on the lipid content of salmon, mackerel, seabass, seabream, sardines, herring, and cod.
