# Supplementary material for: Cardio-Protective Properties and Health Benefits of Fish Lipid Bioactives; The Effects of Thermal Processing
Source: Mar Drugs. 2022 Mar 2;20(3):187. doi: 10.3390/md20030187 (PMC8955125; doi:10.3390/md20030187)
Supplement: Supplementary file 1 [file marinedrugs-20-00187-s001.zip › marinedrugs-1580627 supplementary.pdf]

marine drugs

Supplementary Table 1. Characteristic examples on the fatty acid composition of important fish species (expressed in % of total fatty acids in TL or PL content of raw samples)

	Salmon					Mackerel			Sea bass			Sardines		Herring					Cod		
**Main Fatty Acids Measured**	**TL [247]**	**TL [248]**	**TL [249]**	**PL from fillets [30]**	**PL from heads [9]**	**TL [34]**	**TL [250]**	**TL {71,251]**	**TL [252]**	**TL from farmed [253]**	**TL from wild [253]**	**PL [59, 117]**	**TL [254]**	**TL [231]**	**PL from fillets [9]**	**PL from heads [9]**	**TL (Spawning) [255]**	**TL (Non Spawning) [255]**	**TL (wild) [256]**	**TL [257]**	**TL [207]**
**Myristic Acid (C14:0)**	4.43	3.74	4.88	2.107	1.401	2.10		2.19		3.00	3.90	1.7	7.91	14.2	0.477	3.101	3.56	3.99	1.5	2.2	1.3
**Palmitic Acid (C16:0)**	17.88	17.9	12.72	22.56	1.367	29.40	54.97	14.5	0.12	13.00	15.10	4.5	24.23	15.0	48.74	19.89	19.62	18.35	22.7	4.5	18.3
**Stearic Acid (C18:0)**	4.27	5.18	2.37	6.331	5.732	3.80		7.17	19.46	3.52	3.80	11	5.41	1.4	8.621	5.088	3.54	2.55	6.2	8.8	3.7
**Oleic Acid (C18:1n9)**	36.44	32.36	16.29	13.31	14.16	8.15	1.60	10.5	3.90	24.13	23.28	7.3	6.81	19.2	8.969	13.30	19.72	23.78	8.1	16.3	12.2
**EPA (C20:5n3)**	5.83	5.7	5.72	10.02	10.89	1.35	15.72	4.74	23.16	4.92	0.00	35.7	11.74	7.5	10.12	8.776	7.52	8.74	11.9	31.3	11.0
**DHA**	7.64	7.36	8.53	12.94	36.87	21.6		35.2	5.58	5.37	6.35	38.9	21.29	6.3	16.79	25.62	9.98	8.58	32.3	35.6	38.6

**(C22:6n 3)**						1															
**ALA (C18:3 n-3)**	2.43	0.95	1.1	2.139	1.496	0.80	9.20	0.72	15.1 1	1.57	1.55	N.D.	0.79	6.5	0.269	0.538	0.25	0.41	N.D.	3	0.8
**Linoleic Acid (C18:2n 6)**	10.84	9.64	3.65	6.938	8.822	5.15	9.59	3.46	0.39	15.6	9.45	N.D	2.12	2.6	0.242	1.205	0.97	0.99	1.1	8	1.7
**n6/n3 ratio**	0.7	-	0.8	0.37	0.211	0.4	0.541	0.1	0.1	1.16	1.10	0.02	0.1	-	0.024	0.101	-	-	0.02	0.02	0.1

*Abbreviations: DHA: Docosahexaenoic Acid ; EPA: Eicosapentaenoic Acid ; ALA: Alpha Linolenic Acid; PL: Polar Lipids

Supplementary Table 2: The effects of different thermal processing - cooking techniques on the lipid content of salmon, mackerel, seabass, seabream, sardines, herring and cod

**Thermal Processing - Cooking Method**	**Effect on salmon lipid content**	**Effect on mackerel lipid content**	**Effect on seabass and seabream lipid content**	**Effect on sardines' lipid content**	**Effect on herring lipid content**	**Effect on cod lipid content**
**Boiling**	• no significant difference on EPA and DHA content [257,258]<br>• No increase in the PV and MDA indexes of lipid peroxidation [206]	-	• An increase of the % lipid wet weight, with no significant changes in the SFA were observed by [195]<br>• In contrast, in other studies SFA, MUFA were increased, n-6 PUFA remained the same, n-3 PUFA and especially EPA and DHA were decreased and subsequently the n-6/n-3 PUFA ratio was increased, while the PUFA/SFA was declined [211]<br>• The marker of oxidationn, thiobarbituric acid (TBA) was increased [224]	• Decreased SFA and increased MUFA content [259]	• Reduction of palmitic acid, with no significant effect on the levels of oleic acid, while EPA and DHA levels were reduced [258]<br>• Instead, in other studies no major losses in the amount of the unsaturated fats, without also a reduction on the n-3 PUFA fraction of the total fatty acid content [231]	• Ttotal level of lipid content ans palmitic acid were decreased, wherease MUFA, especially oleic acid, were increased, while the amounts of n-3 and n-6 PUFA did not alter and the n-6/n-3 PUFA was slightly increased upon thermal treatment [259]

Thermal Processing - Cooking Method	Effect on salmon lipid content	Effect on mackerel lipid content	Effect on seabass and seabream lipid content	Effect on sardines' lipid content	Effect on herring lipid content	Effect on cod lipid content
**Steaming**	• no significant differences in MUFA (oleic acid), DHA, EPA, DPA and overall lipid content, as well as in lipid oxidation [210,248,261]<br>• On the other hand, in other studies n-3 PUFA were increased [247]<br>• Instead, other studies reported slight reduction on the n-3 PUFA (EPA and DHA) content was reported [257], as well as decreased cholesterol content [261]	• Total lipid content, n6/n3 PUFA ratio and Vitamin A were decreased, while oleic acid, overall MUFA, PUFA, and especially n-3 PUFA and Vitamin D3 were increased [34]<br>• Other studies reported Increased PUFA and n-3 PUFA, while MUFA were decreased [247]<br>• In contrast, others reported a substantial decrease in the content of DHA and loss of Docosadienoic acid [250], but an increase in the PL [262,263], and an increase in the Peroxide and thiobarbituric acid values	• An increase of the % lipid wet weight, with no significant changes in the SFA [195]<br>• The marker of oxidationn, thiobarbituric acid (TBA) was also increased [224]	•	•	• A decrease of fat content a very slight increase in SFAs and MUFAs, while diminishing the PUFA amount. for vitamin D3 steaming retains the most Vitamin E was relatively stable during steaming [233]

**Thermal Processing - Cooking Method**	**Effect on salmon lipid content**	**Effect on mackerel lipid content**	**Effect on seabass and seabream lipid content**	**Effect on sardines' lipid content**	**Effect on herring lipid content**	**Effect on cod lipid content**
**Oven Cooked / Baking / Roasting**	• No significant changes in SFA, MUFA or PUFA % content [248]<br>• On the other hand others reported increased PUFA, with a slight increase of n-3 PUFA, EPA and DHA [247], while cholesterol was decreased [261]<br>• In contrast others reported that n-3 PUFA (EPA and DHA) were slightly reduced [257,258]<br>• Others found that MUFA and DHA were increased, with decreased overall n-3 PUFA content and PUFA were not significantly oxidised [264].<br>• Changes in PUFA lev-	• no drastic changes in the SFA composition, MUFA were decreased, while PUFA and especially the n-6 PUFA were increased [247].	• SFA, MUFA were increased, n-6 PUFA remained the same, n-3 PUFA and especially EPA and DHA were decreased and subsequently the n-6/n-3 PUFA ratio was increased, while the PUFA/SFA was declined [211].<br>• On the other hand others reported that during oven Broiling, both MUFA and SFA were decreased [265]	• The fatty acid profile and the n-3/n-6 PUFA ratio has not been substantially changed, even though a slight increase in the EPA and DHA levels was observed [254]	• No changes in the lipid abundance and composition and the n-6/n-3 PUFA ratio and overall content of PUFA [266]	• Most of the lipids were preserved for both wild and farmed cod [256,266]<br>• Instead others reported a decrease of fat content, with a very slight increase in SFAs and MUFAs, while diminishing the PUFA content, while vitamin E remained relatively stable [233]

Thermal Processing - Cooking Method	Effect on salmon lipid content	Effect on mackerel lipid content	Effect on seabass and seabream lipid content	Effect on sardines' lipid content	Effect on herring lipid content	Effect on cod lipid content
	els were accompanied with increased of lipid peroxidation indexes, PV and MDA[206]<br>• In contrast others reported no significant differences and oxidation of lipids, during baking[260]					
**Grilled**	• No significant difference on the EPA and DHA content [257,258]<br>• In contrast, others reported that the fatty acid content, and especially the n-3 PUFA, EPA and DHA, were increased, with stability against oxidation, while cholesterol was decreased [260,261].	• Large increase in total lipid content, associated with increases in SFA, while MUFA and PUFA (especially DHA), were decreased, without however affecting significantly the n-6/n-3 ratio [250,262]<br>• Heptadecanoic, arachidonic and Docosadienoic acid were lost after grilling [250]	• No changes in the total lipid content [267]<br>• In contrast, others reported that levels of SFA and MUFA were decreased, PUFA were increased , while losses in n-3 PUFA content were observed, as well as reduction of myristic acid, palmitic acid, palmitoleic acid, stearic acid, elaidic acid, oleic acid, linoleic acid, henicosanoic	• Reduced lipid content and decrease in the SFA, MUFA and PUFA [268], with a high loss of n-3 PUFA, such as EPA and DHA [259], due to the autoxidation of lipids, forming atty acyl hydroperoxides [268]<br>• PL from the sardines undergone to heat treatment during grilling were 8 times less biologically ac-	• No major losses in the amount of the unsaturated fats, without also a reduction on the n-3 PUFA fraction of the total fatty acid content [231]	• A significant increase was observed in the n-6/n-3 PUFA ratio [266]

Thermal Processing - Cooking Method	Effect on salmon lipid content	Effect on mackerel lipid content	Effect on seabass and seabream lipid content	Effect on sardines' lipid content	Effect on herring lipid content	Effect on cod lipid content
			acid, eicosenoic acid, EPA, nervonic acid and DHA fatty acid contents [252]<br>• Others have also reported that grilling did not affect the cardio-protective bioactivities of sea bream PL against the platelet aggregation induced by the highly bioactive inflammatory mediator platelet activating factor (PAF) [232]	tive, against the inflammatory mediator PAF, than the raw sample [117]		
**Pan frying without the addition of fat/oil**	• No significant changes in SFA, MUFA or PUFA % content [248,210], while the n-6/n-3 PUFA ratio was increased [210]<br>• Others have reported significant change in PUFA levels, with the	•	•	• Altered fatty acids content, with increased oleic and linoleic acids and lower EPA and DHA levels [250]	•	• A significant increase was observed in the n-6/n-3 PUFA ratio [266]

Thermal Processing - Cooking Method	Effect on salmon lipid content	Effect on mackerel lipid content	Effect on seabass and seabream lipid content	Effect on sardines' lipid content	Effect on herring lipid content	Effect on cod lipid content
	peroxidation of both n-3 PUFA and n-6 PUFA being intensified, with a subsequent increase in the PV and MDA indexes of lipid peroxidation, and significant elevation in 4-HHE and 4-HNE levels [206].					
**Pan-frying in extra virgin Olive Oil**	• Higher contents of MUFA, with slight increase in oleic acid and SFA [269], and slightly (but not significantly) decreased EPA and DHA and increased n-6/n-3PUFA ratio [208,264], while PUFA were not significantly oxidised [264]	•	•	• Increased MUFA, especially oleic acid, decreased SFA and PUFA, with reduced n-3 PUFA, especially EPA and DHA, while n-6 PUFA were increased, especially linoleic acid [269,270]	•	• Increased MUFA and decreased SFA and PUFA, while individually palmitic acid, oleic acid, linoleic acid, DHA and EPA levels, with the n-6/n-3 ratio being increased [207]<br>• Instead others reported increased overall fatty acids' content, SFA, MUFA and PUFA (especially

**Thermal Processing - Cooking Method**	**Effect on salmon lipid content**	**Effect on mackerel lipid content**	**Effect on seabass and seabream lipid content**	**Effect on sardines' lipid content**	**Effect on herring lipid content**	**Effect on cod lipid content**
						oleic acid and linoleic acid, while significant decreases were reported for palmitic, α-linolenic, DHA and EPA), with a subsequent slight increase of the n-6/n-3 PUFA ratio [208]
**Pan-frying in Sunflower oil**	• Decreased EPA and DHA [258], while the n-6 linoleic acid was increased, and thus the n-6/n-3PUFA ratio was increased [208]	• Increased total lipid content and oleic acid, PUFA levels and the n6/n3 PUFA ratio, wherase SFA were decreased [34].<br>• Reduced vitamin A and significant increase in vitamin E (α-tocopherol)[34]	• Increased lipid content [267]<br>• Increased MUFA and n-6 PUFA, while SFA and n-3 PUFA (especially DHA and EPA) were decreased, with a subsequent incresе in the n-6/n-3 PUFA and the PUFA/SFA ratios [211]	• Increased oleic acid and decreased SFA and n-3 PUFA (especially EPA and DHA), while n-6 PUFA were increased [269]	• Reduction in the levels of palmitic acid, with no significant effect on the levels of oleic acid, while EPA and DHA levels were reduced [258]	• increased overall fatty acids' content, SFA, MUFA and PUFA (especially oleic acid and linoleic acid, while significant decreases were reported for palmitic, α-linolenic, DHA and EPA), with a subsequent slight increase of the n-6/n-3 PUFA ratio [208]<br>• PL retained their strong an-

Thermal Processing - Cooking Method	Effect on salmon lipid content	Effect on mackerel lipid content	Effect on seabass and seabream lipid content	Effect on sardines' lipid content	Effect on herring lipid content	Effect on cod lipid content
						ti-inflammatory and anti-atherogenic properties against the PAF-pathways, while in some cases this anti-inflammatory potency was even increased [118].
**Pan Frying with Corn OIl**	• Slight exchange of fats between the pan-frying medium and the sample, and a slight increase in the SFA. [210]	•	• Increased MUFA and n-6 PUFA and decreased SFA and n-3 PUFA (especially DHA and EPA), with a subsequent increase in the n-6/n-3 PUFA and PUFA/SFA ratios [211]	•	•	•
**Pan Frying with Soya OIl**	• No significant differences in the fatty acid profile, but the n-6/n-3 PUFA ratio was increased [264]	•	•	•	•	•
**Pan Frying with palm**	• A slight increase in the SFA.	• Changes in the SFA/PUFA ratio, with	•	•	•	•

Thermal Processing - Cooking Method	Effect on salmon lipid content	Effect on mackerel lipid content	Effect on seabass and seabream lipid content	Effect on sardines' lipid content	Effect on herring lipid content	Effect on cod lipid content
**oil**	• Partially hydrogenated plant oil did not increase the trans fatty acids content [210]	losses of the heptadecanoic, elaidic, erucic and arachidonic acid, accompanied with a significant decrease of EPA and DHA [250]				
**Pan Frying with Coconut Oil**		• Highly increased SFA (418%),, while MUFA were also increased and PUFA were decreased (especially EPA and DHA) [271]	•	•	•	•
**Other types of frying (i.e. deep frying or frying with rapeseed oil or margarine or lard or "Smazyk")**	• Deep frying induced increased SFA and decreased MUFA, but oleic acid was increased, while linoleic acid was retained, whereas DHA, EPA and DPA were reduced [248]	•	•	• Frying with lard did not affect n-3 PUFA, while oleic acid and n-6 PUFA were increased [270]	• Frying with "Smazyck" showed no major losses in the amount of the unsaturated fats and especially in n-3 PUFA [231]	• Frying with margarine increased lipid content and SFA, while PUFA were decreased and the n-6/n-3 PUFA ratio was increased in [207]<br>• Frying in rapeseed oil increased also the fat content and MUFA, and reduced SFA and PUFA, while vitamin A (retinol) was re-

Thermal Processing - Cooking Method	Effect on salmon lipid content	Effect on mackerel lipid content	Effect on seabass and seabream lipid content	Effect on sardines' lipid content	Effect on herring lipid content	Effect on cod lipid content
						tained and vitamin E was increased [233]
**Sous-Vide**	• The n-3 PUFA content, the n-6/n-3 PUFA ratio and the anti-inflammatory and antithrombotic biofunctionality of PL baring n-3 PUFA were retained in low temperatures [57].		• Increased lipid content, with no significant changes in the SFA [195]<br>• The marker of oxidationn, thiobarbituric acid (TBA) was increased, but this increase was much lower than boiled or steamed samples, suggesting much less oxidation in sous vide heat treatment [224]	•	•	•

Abbreviations: EPA = eicosapentaenoic acid; TFA = trans fatty acids; DHA = docosahexaenoic acid; DPA = docosapentaenoic acid; MUFA- monounsaturated fatty acids; FA = fatty acids; PUFA- polyunsaturated fatty acids; SFA- saturated fatty acids;
